# Supplementary material for: What/Why/When/Where/How Framework and Faculty Development Workshop to Improve the Utility of Narrative Evaluations for Assessing Internal Medicine Residents
Source: MedEdPORTAL. 2024 Jul 30;20:11420. doi: 10.15766/mep_2374-8265.11420 (PMC11286767; doi:10.15766/mep_2374-8265.11420)
Supplement: Supplementary file 1 — Workshop Slides.pptxFramework.docxMock Learner Video 1.mp4Mock Learner Video 2.mp4Surveys.docxUtility Grading Rubric.docxFacilitator Guide.docx [file mep_2374-8265.11420-s001.zip › E. Surveys.docx]

**Appendix E: Surveys**

These surveys were used to assess confidence of our participants pre and post-intervention. These surveys are not intended to be used as part of the workshop. However, other institutions may wish to use these surveys to gauge their own faculty’s’ confidence in these skills.

Faculty Participant Survey – Pre-Intervention

| Questions | | |  | | | | | | | | | | | | | |  | | | |
| --- | --- | --- | --- | --- | --- | --- | --- | --- | --- | --- | --- | --- | --- | --- | --- | --- | --- | --- | --- | --- |
| Have you had prior training in improving written evaluations? | | |  | | | | | | | | | Yes | | | |  | | | | No |
| How many years have you been in a faculty supervisory role of residents? | | | 0-5 | | | | | 5-10 | | | | | | | | >10 | | | | |
| In which Division are you faculty? | Cardiology | Pulmonary or CCM | | | GIM | | Hospitalist | | ID | | | Renal | | | Endo | | | Rheum | | Heme/ Onc |
| In which setting do you supervise residents? | Primary Care Clinic | | | Subspecialty Clinic | | | Medicine Wards | | | | Cardiology Ward/Unit | | | | ICU | | | | | Subspecialty Consults |
|  | | | Program Leadership | | | | | | | | | | Resident | | | | | | Both | |
| Which audience do you consider when writing the evaluation? | | |  | | | | | | | | | |  | | | | | |  | |
|  | | | 1 Not at all confident | | | 2 Not very confident | | | | 3 Neutral | | | | 4 Somewhat confident | | | | | | 5 Very confident |
| How confident in your **overall ability** to write a high-quality written evaluations? | | |  | | |  | | | |  | | | |  | | | | | |  |
| How confident are you in your ability to write an evaluation that is useful for the **resident**? | | |  | | |  | | | |  | | | |  | | | | | |  |
| How confident are you in your ability to write an evaluation that is useful for **program leadership** in assessing the resident? | | |  | | |  | | | |  | | | |  | | | | | |  |
| How confident are you in your ability to providing **specific behaviors** that reflect trainee performance? | | |  | | |  | | | |  | | | |  | | | | | |  |
| How confident are you in your ability to providing appropriate **context** of the behavior (type of patient, patient setting, trainee year of training)? | | |  | | |  | | | |  | | | |  | | | | | |  |
| How confident are you in your ability to providing comments on **change in trainee performance over time?** | | |  | | |  | | | |  | | | |  | | | | | |  |
| Is there anything else you find difficult in writing and evaluation? | | |  | | | | | | | | | | | | | | | | | |

Faculty Participant Survey – Post-Intervention

| Which audience do you consider when writing the evaluation? | Program Leadership | | | Resident | | | | Both | | |
| --- | --- | --- | --- | --- | --- | --- | --- | --- | --- | --- |
|  | 1 Not at all confident | 2 Not very confident | | | 3 Neutral | | 4 Somewhat confident | | | 5 Very confident |
| How confident are you in your **overall ability** to write a high-quality written evaluations? |  |  | | |  | |  | | |  |
| How confident are you in your ability to write an evaluation that is useful for the **resident**? |  |  | | |  | |  | | |  |
| How confident are you in your ability to write an evaluation that is useful for **program leadership** in assessing the resident? |  |  | | |  | |  | | |  |
| How confident are you in your ability to providing **specific behaviors** that reflect trainee performance? |  |  | | |  | |  | | |  |
| How confident are you in your ability to providing appropriate **context** of the behavior (type of patient, patient setting, trainee year of training)? |  |  | | |  | |  | | |  |
| How confident are you in your ability to providing comments on **change in trainee performance over time?** |  |  | | |  | |  | | |  |
| Please comment further on your planned change: |  | | | | | | | | | |
| Which portion of the session did you find most useful? Please rank the following. | Framework | | Explanations of key features that are helpful to the CCC | | | Avoiding Hedging | | | Importance of specificity | |
| Is there anything you would change about this workshop? |  | | | | | | | | | |

Faculty Participant Survey – 3 months Post-Intervention

| Please answer the questions below about your experience in writing written evaluations of residents since completing the Faculty Workshop on Written Evaluations about 3 months ago. | | | | | | | | | | |
| --- | --- | --- | --- | --- | --- | --- | --- | --- | --- | --- |
| Which audience do you consider when writing the evaluation? | Program Leadership | | | Resident | | | | Both | | |
|  | 1 Not at all confident | 2 Not very confident | | | 3 Neutral | | 4 Somewhat confident | | | 5 Very confident |
| How confident are you in your **overall ability** to write a high-quality written evaluations? |  |  | | |  | |  | | |  |
| How confident are you in your ability to write an evaluation that is useful for the **resident**? |  |  | | |  | |  | | |  |
| How confident are you in your ability to write an evaluation that is useful for **program leadership** in assessing the resident? |  |  | | |  | |  | | |  |
| How confident are you in your ability to providing **specific behaviors** that reflect trainee performance? |  |  | | |  | |  | | |  |
| How confident are you in your ability to providing appropriate **context** of the behavior (type of patient, patient setting, trainee year of training) |  |  | | |  | |  | | |  |
| How confident are you in your ability to providing comments on **change in trainee performance over time?** |  |  | | |  | |  | | |  |
| Have you changed your approach to writing written evaluations of residents? | Yes | | | | | No | | | | |
| Do you use the ABCD framework when writing written evolutions of residents? | Yes | | | | | No | | | | |
| Now that you have had time to practice the workshop skills, which portion of the session did you find most useful? | Framework | | Explanations of key features that are helpful to the CCC | | | Avoiding Hedging | | | Importance of specificity | |
| Now that you have had time to practice the workshop skills, is there anything you would change about this workshop? |  | | | | | | | | | |
