# Supplementary material for: What/Why/When/Where/How Framework and Faculty Development Workshop to Improve the Utility of Narrative Evaluations for Assessing Internal Medicine Residents
Source: MedEdPORTAL. 2024 Jul 30;20:11420. doi: 10.15766/mep_2374-8265.11420 (PMC11286767; doi:10.15766/mep_2374-8265.11420)
Supplement: Supplementary file 1 — Workshop Slides.pptxFramework.docxMock Learner Video 1.mp4Mock Learner Video 2.mp4Surveys.docxUtility Grading Rubric.docxFacilitator Guide.docx [file mep_2374-8265.11420-s001.zip › F. Utility Grading Rubric.docx]

**Appendix F: Utility Grading Rubric**

Other institutions may wish to use this rubric in assessing how well their faculty write narrative evaluations that are useful for clinical competency committees. This may also be used as a self-reflective tool for ongoing faculty development, as described in the discussion of the manuscript.

| **Question** | **1** **Not Useful** | **2 Somewhat Useful** | **3 Moderately Useful** | **4 Extremely Useful** |
| --- | --- | --- | --- | --- |
| In answering the questions below, please consider whether the written evaluation is *useful in assessing residency competency* such that you are able to make a decision regarding promotion or remediation | | | | |
| For the purpose of making a promotion or coaching decision of this resident, how useful is… | | | | |
| … the description of **specific behaviors of strengths** provided in this evaluation? | Not mentioned | Gives a judgment (“is a good resident”) | Names the strength (“excellent fund of knowledge”) | Gives an example (“manages ADHF”) |
| …the description of **specific behaviors of weaknesses (areas for growth)** provided in this evaluation? | Not mentioned | Gives a judgment (“poor team leader”) | Names the problem (“rounds are disorganized”) | Gives an example (“spends too much time teaching”) |
| … the **context of clinical environment** (patient acuity, busy service, etc) to appropriately assess competency? | Not mentioned | Can discern e.g. in- vs out-patient, or walk rounds vs afternoon teaching | Gives learner’s level, and/or patient acuity, team size, etc. (“appropriate for an early 2^nd^-year resident”) | Gives learner’s level AND patient acuity, team size, etc. (“managing a busy inpatient service with frequent turnover like a hospitalist”) |
| … the description of **response to feedback/improvement overtime** | Not mentioned | Notes presence/absence of response (“doesn’t accept feedback well”) | Describes response (“improves with feedback”) | Gives an example (“gave more organized assessments after we discussed it”) |
| …the description of **actionable next steps** for improvement | Not mentioned | Identifies need for improvement (“will improve with practice”) | Names the problem (“should spend less time teaching on rounds”) | Gives an example (“will prepare teaching points that last 1-3 minutes before rounds”) |

**Development:**

Components of our grading rubric included an overall score as well as components of the framework: behaviors of strength, behaviors of growth, context, improvement over time, and actionable next steps. The items and scale of the adapted graded rubric were discussed and optimized iteratively with a focus group of 4 members of the internal medicine CCC, resulting in 6 items on a 5-pt Likert scale. Then, two medicine-pediatric residency CCC members scored 6 randomly selected sample narrative evaluations and discussed for consensus. This discussion resulted in the final grading rubric with the same 6 items but on a 4-pt Likert scale, for a total score of 24. We assessed the rubric for inter-rater reliability using inter-class co-coefficients based on all analyzed MEs.
