# Supplementary material for: What/Why/When/Where/How Framework and Faculty Development Workshop to Improve the Utility of Narrative Evaluations for Assessing Internal Medicine Residents
Source: MedEdPORTAL. 2024 Jul 30;20:11420. doi: 10.15766/mep_2374-8265.11420 (PMC11286767; doi:10.15766/mep_2374-8265.11420)
Supplement: Supplementary file 1 — Workshop Slides.pptxFramework.docxMock Learner Video 1.mp4Mock Learner Video 2.mp4Surveys.docxUtility Grading Rubric.docxFacilitator Guide.docx [file mep_2374-8265.11420-s001.zip › G. Facilitator Guide.docx]

**Appendix G Facilitator Guide**:

Please use this Facilitator Guide to guide the use of the workshop materials.

Setting:

The original implementation was conducted in lecture style. However, it can be adapted to small group discussion around the case videos provided.

Participants:

This content was developed for faculty that supervise internal medicine residents. However, it may be adapted for other subspecialities for which hospital-based care and clinic-base care are the primary settings.

Agenda:

- Why written narrative evaluations are important (8 min)
- Introduce the idea of actionable feedback (8 min)
- Case practice for priming (4 minute video, 2 minutes to write)
- Review the Framework (15 min)
- Case practice for deliberate practice (4 minute video, 2 minutes to write)
- Tools (8 min)

How to use the slides:

- We have provided notes on each slide for talking points. Please adapt these points to what is appropriate to your institution
- Recommended script is noted in quotations. Other text describe recommended talking points.

How to use the videos:

- The videos were designed for priming and deliberate practice of the provided why/what/when/where framework during the powerpoint-based workshop. Participants will watch the videos as indicated in the power point and have time to write narratives.
- You may decide to adapt their use to small groups where participants can review each other’s narratives and discuss different approaches for observation and narrative verbiage.
