# Supplementary material for: What/Why/When/Where/How Framework and Faculty Development Workshop to Improve the Utility of Narrative Evaluations for Assessing Internal Medicine Residents
Source: MedEdPORTAL. 2024 Jul 30;20:11420. doi: 10.15766/mep_2374-8265.11420 (PMC11286767; doi:10.15766/mep_2374-8265.11420)
Supplement: Supplementary file 1 — Workshop Slides.pptxFramework.docxMock Learner Video 1.mp4Mock Learner Video 2.mp4Surveys.docxUtility Grading Rubric.docxFacilitator Guide.docx [file mep_2374-8265.11420-s001.zip › A. Workshop Slides.pptx]

## Slide 1
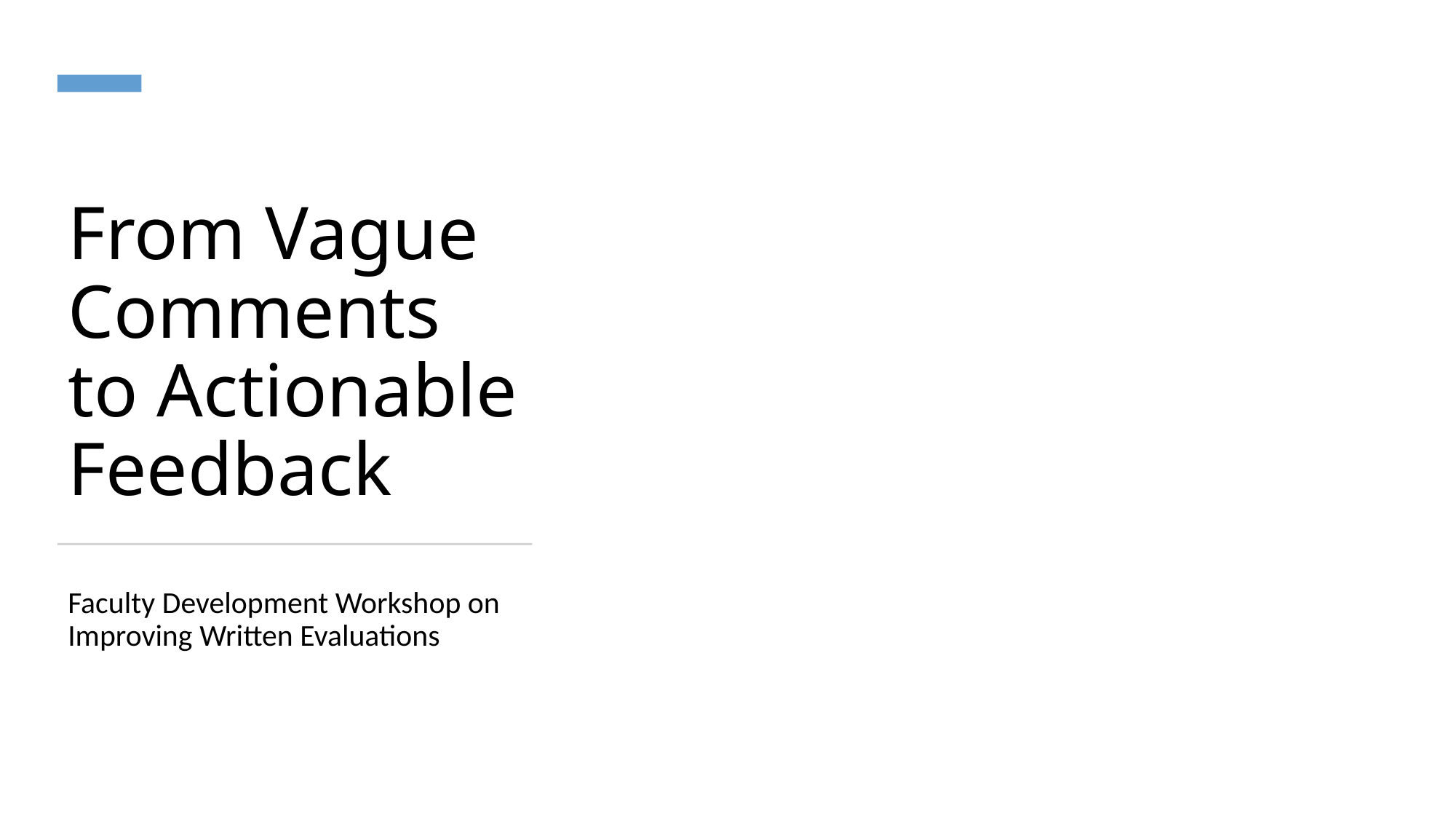

# From Vague Comments to Actionable Feedback
Faculty Development Workshop on Improving Written Evaluations

## Slide 2
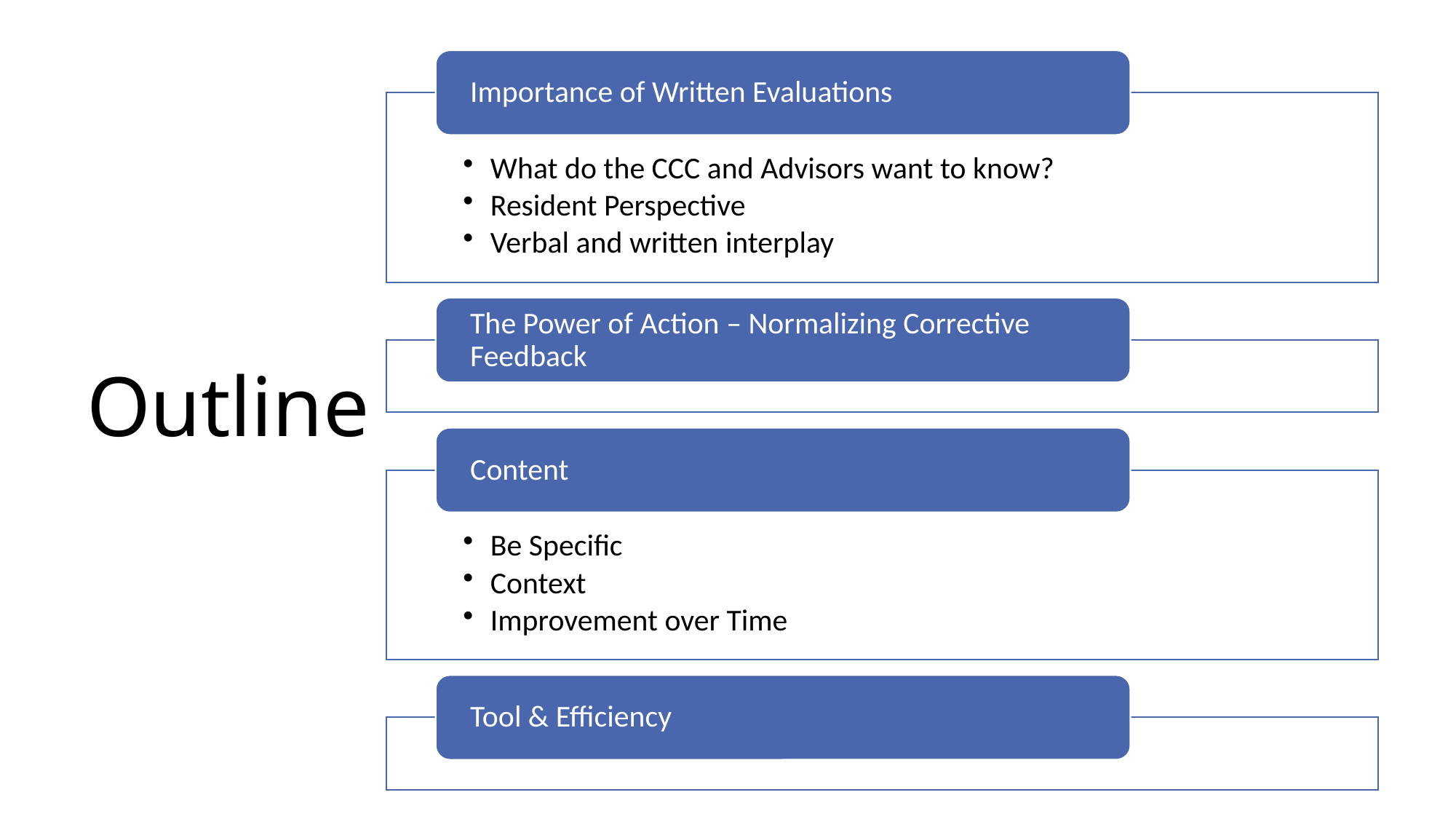

# Outline

## Slide 3
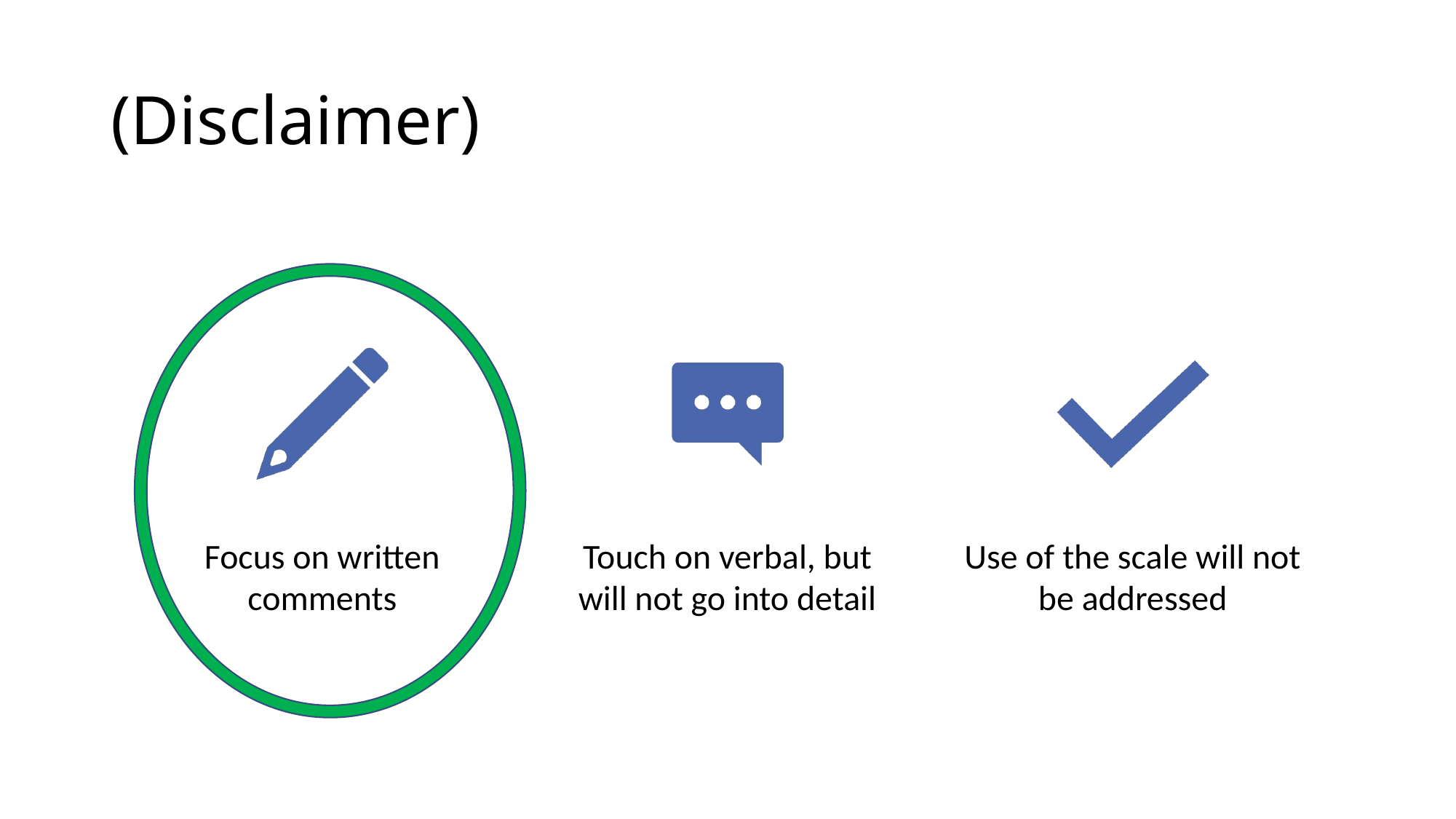

# (Disclaimer)

## Slide 4
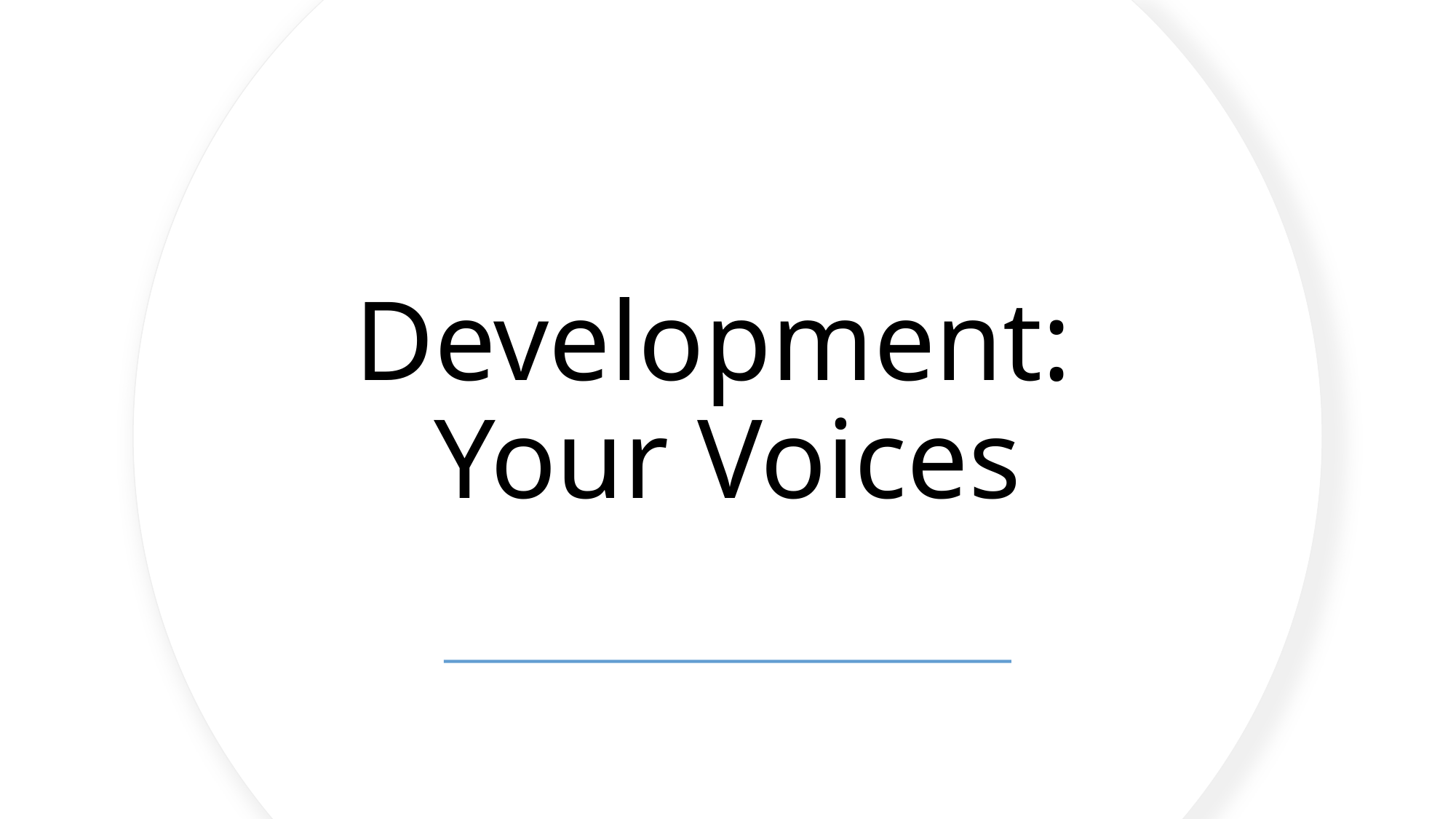

# Development: Your Voices

## Slide 5
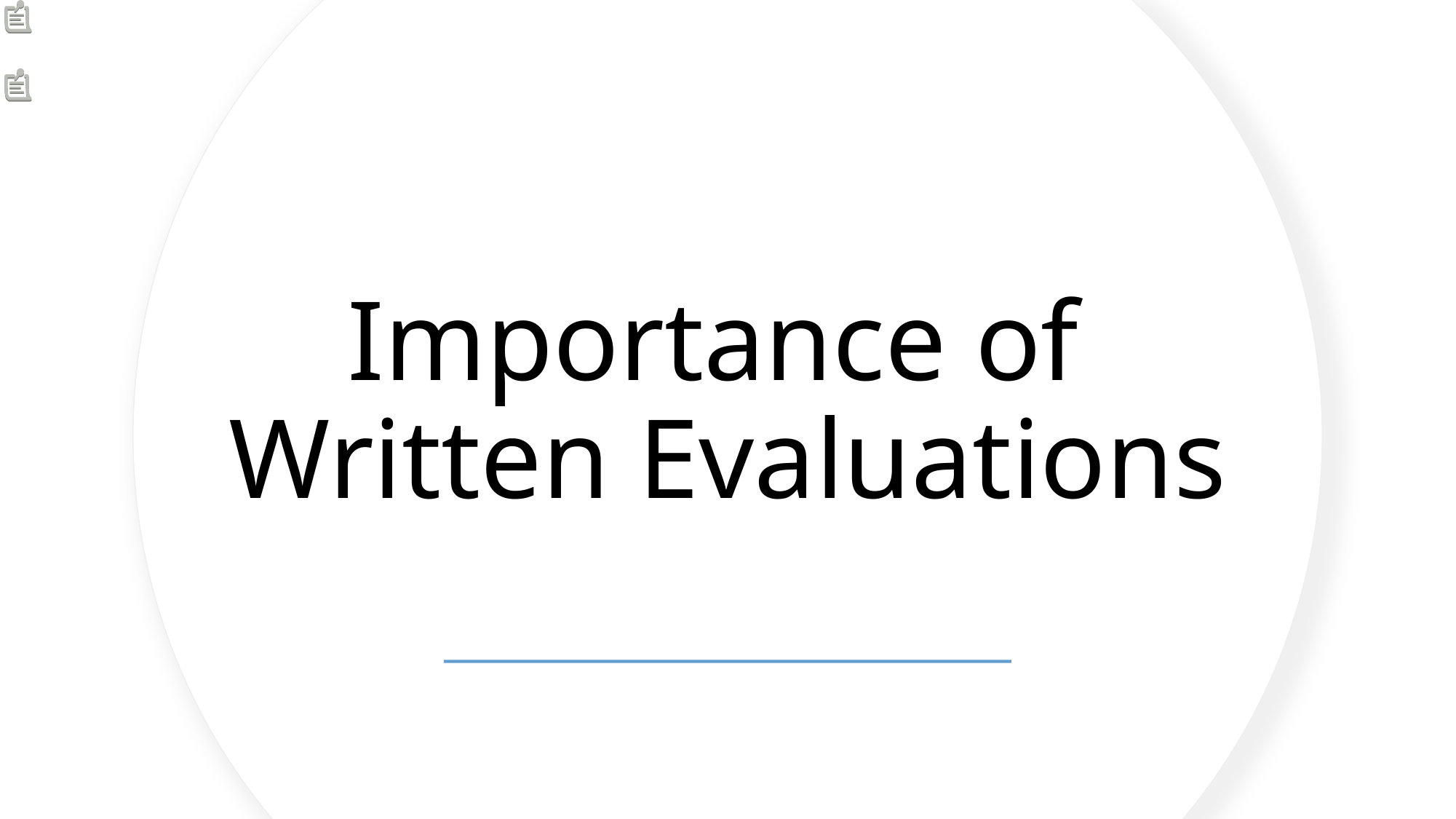

# Importance of Written Evaluations

## Slide 6
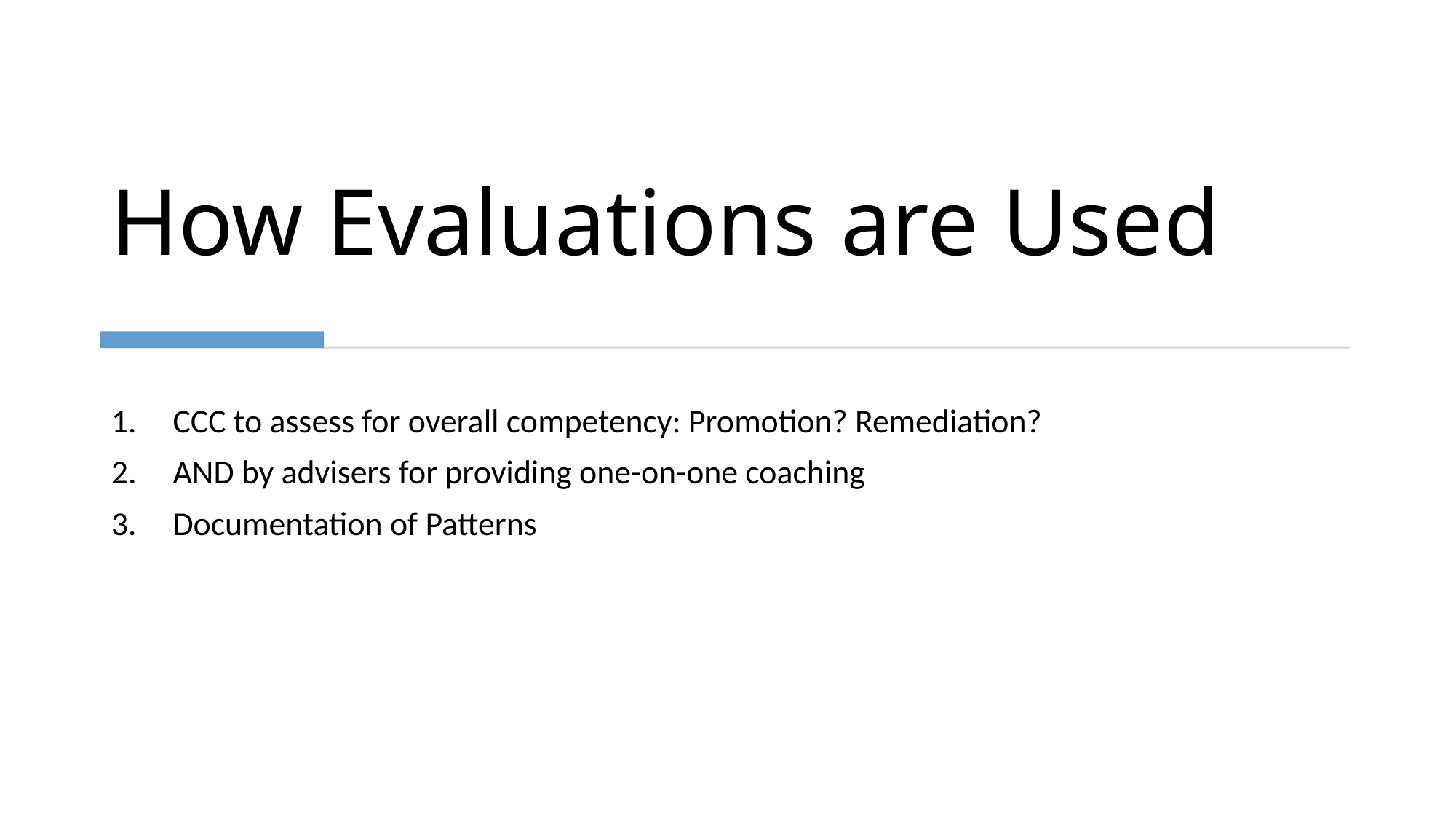

# How Evaluations are Used
CCC to assess for overall competency: Promotion? Remediation?
AND by advisers for providing one-on-one coaching
Documentation of Patterns

## Slide 7
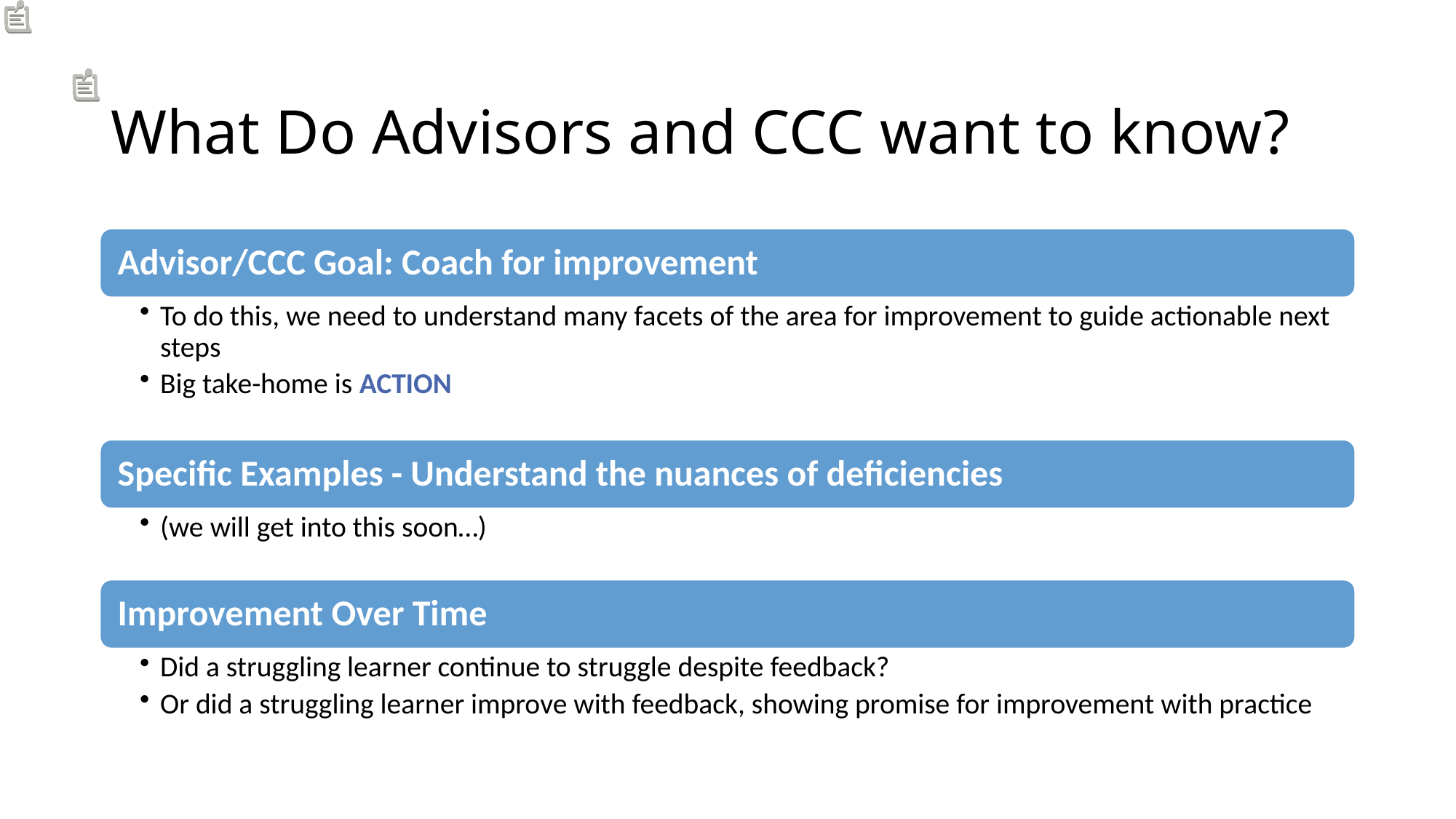

# What Do Advisors and CCC want to know?

## Slide 8
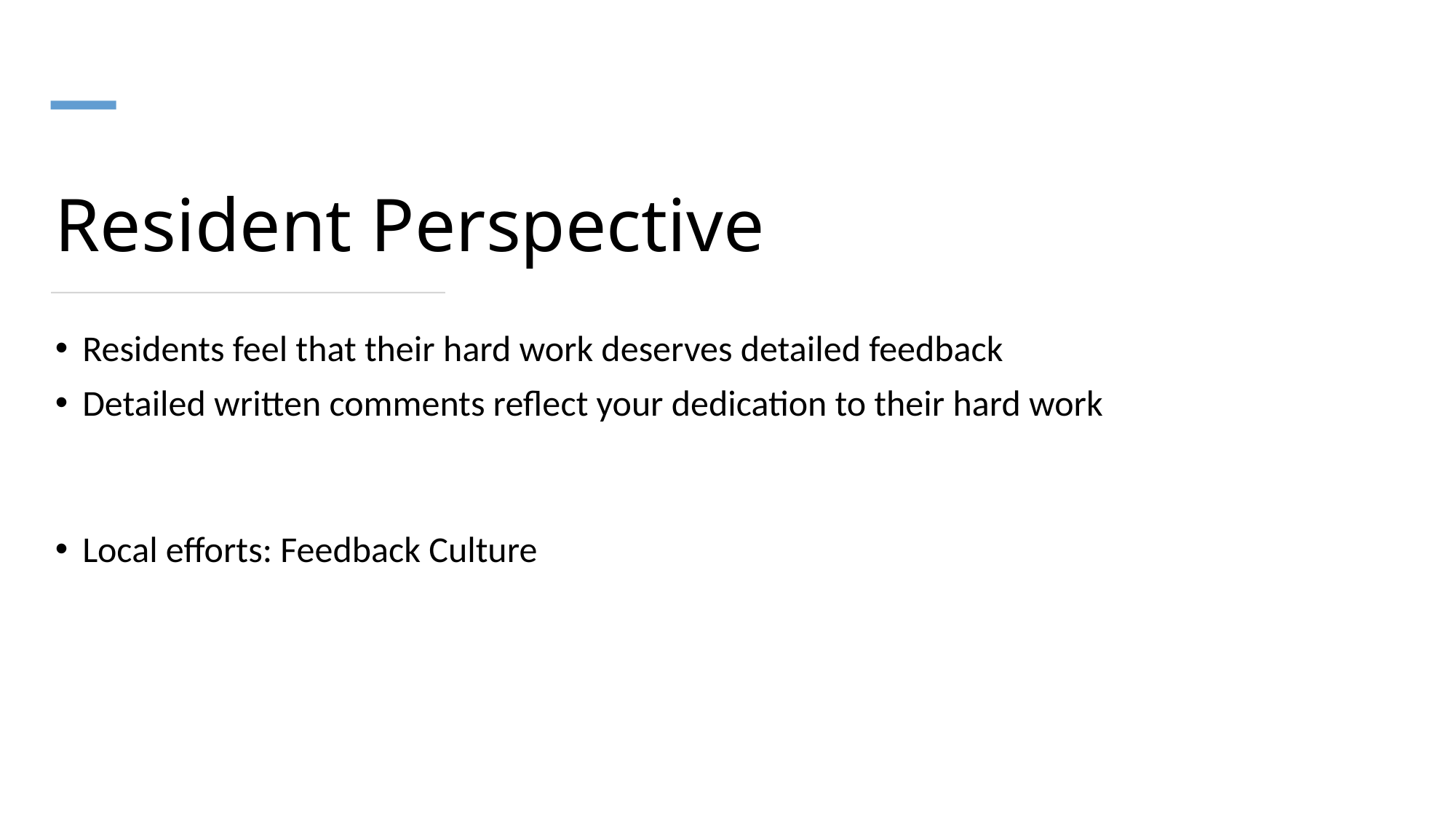

# Resident Perspective
Residents feel that their hard work deserves detailed feedback
Detailed written comments reflect your dedication to their hard work
Local efforts: Feedback Culture

## Slide 9
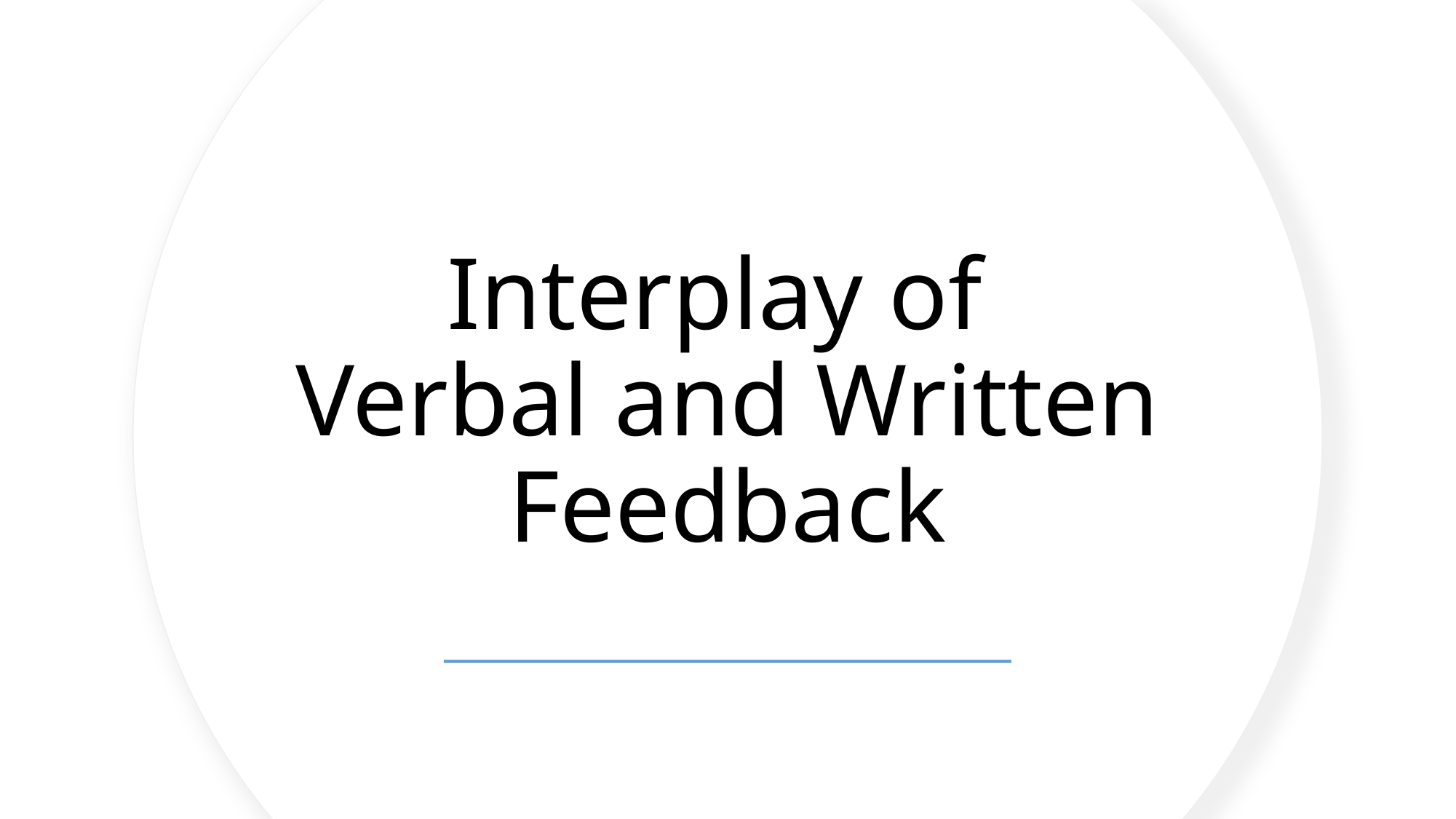

# Interplay of Verbal and Written Feedback

## Slide 10
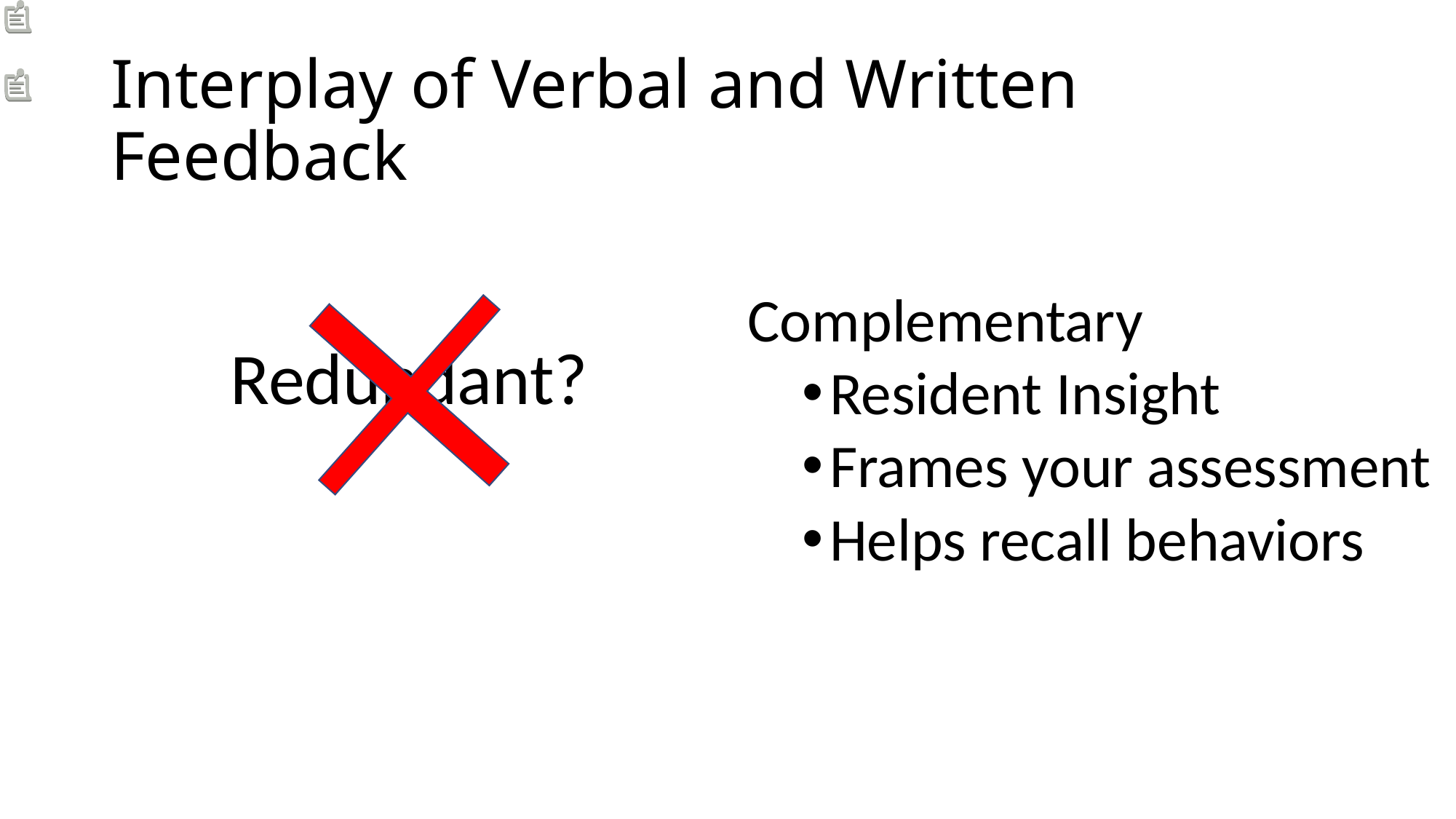

# Interplay of Verbal and Written Feedback
Complementary
Resident Insight
Frames your assessment
Helps recall behaviors
Redundant?

## Slide 11
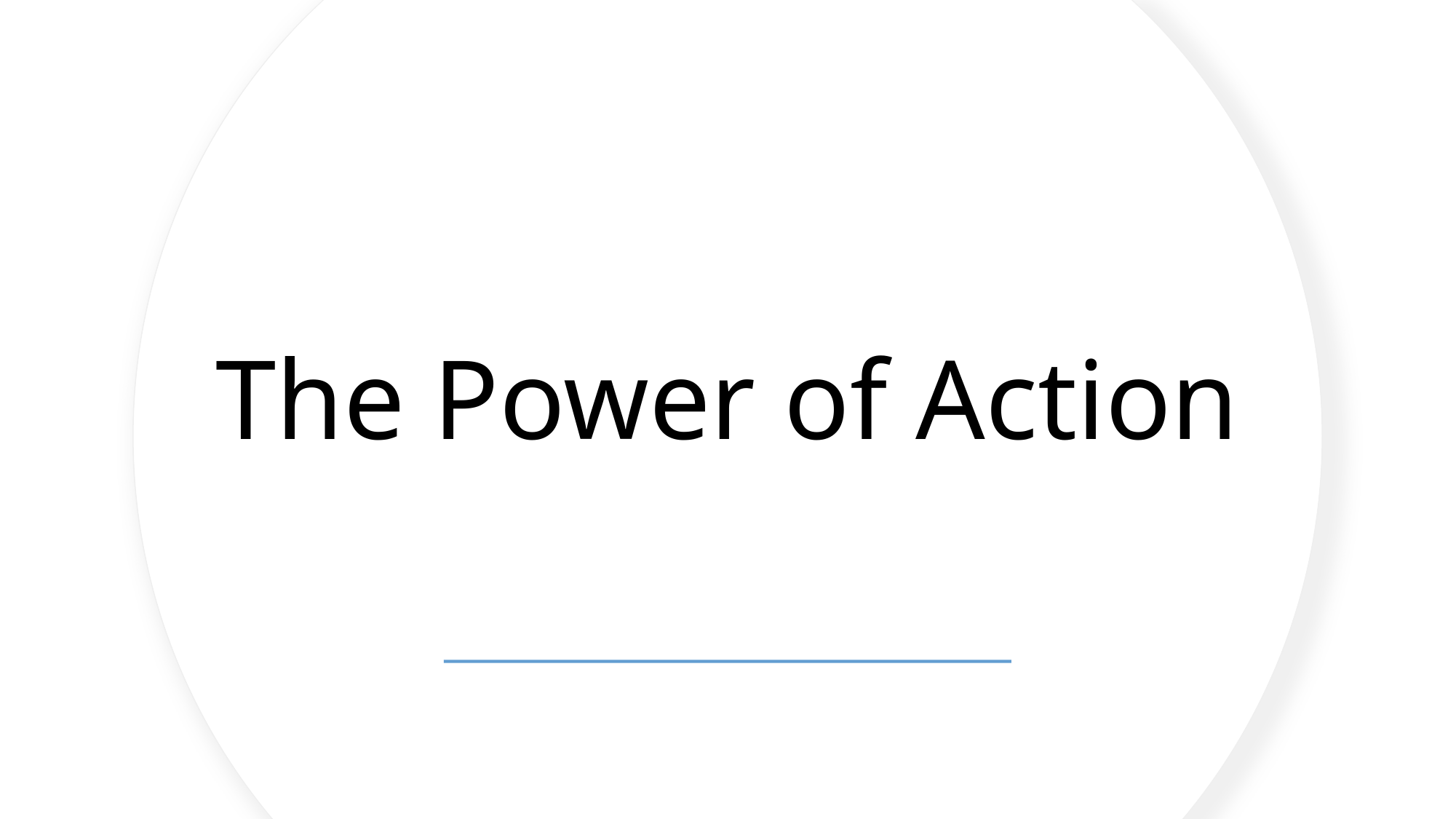

# The Power of Action

## Slide 12
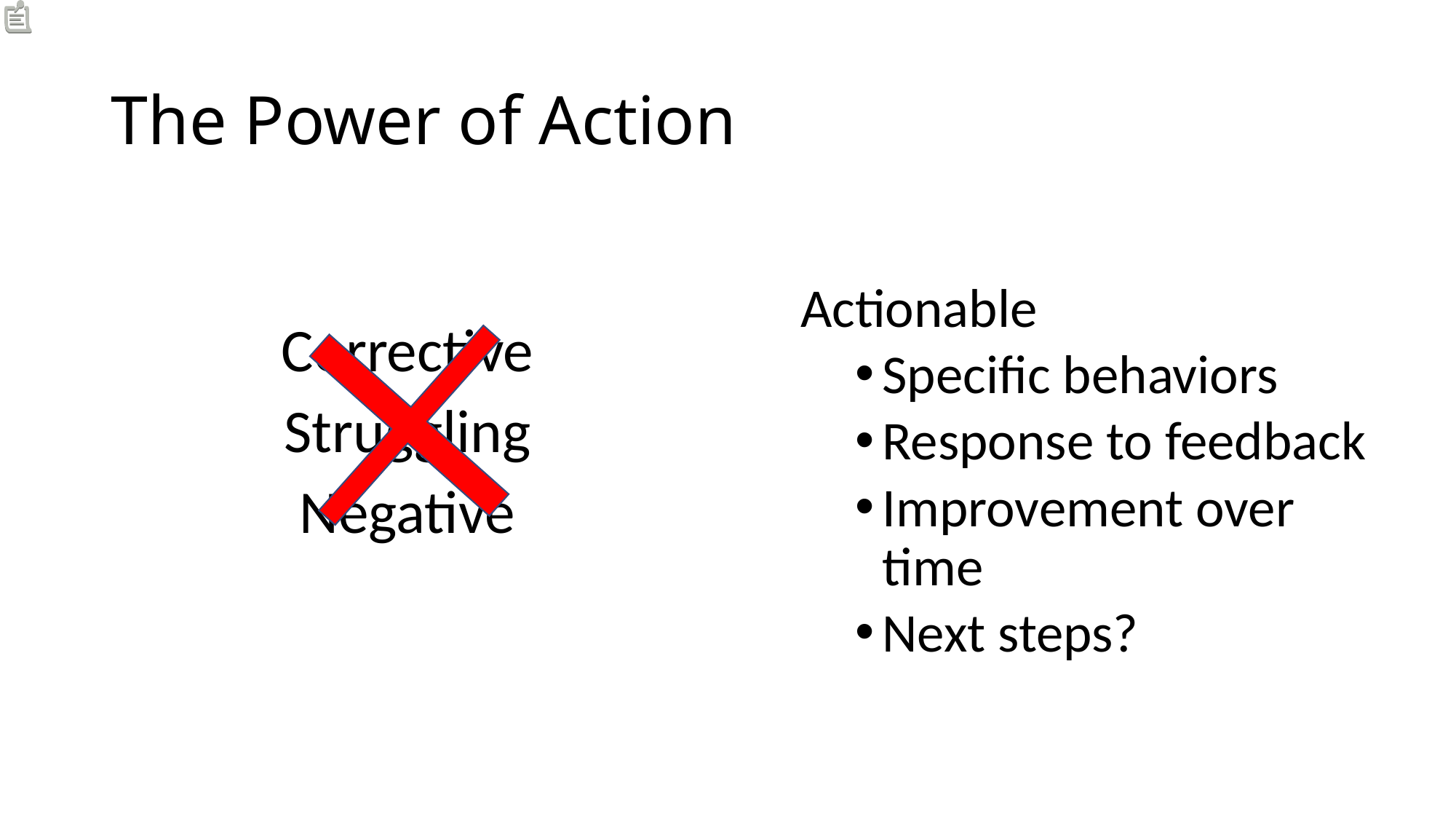

# The Power of Action
Actionable
Specific behaviors
Response to feedback
Improvement over time
Next steps?
Corrective
Struggling
Negative

## Slide 13
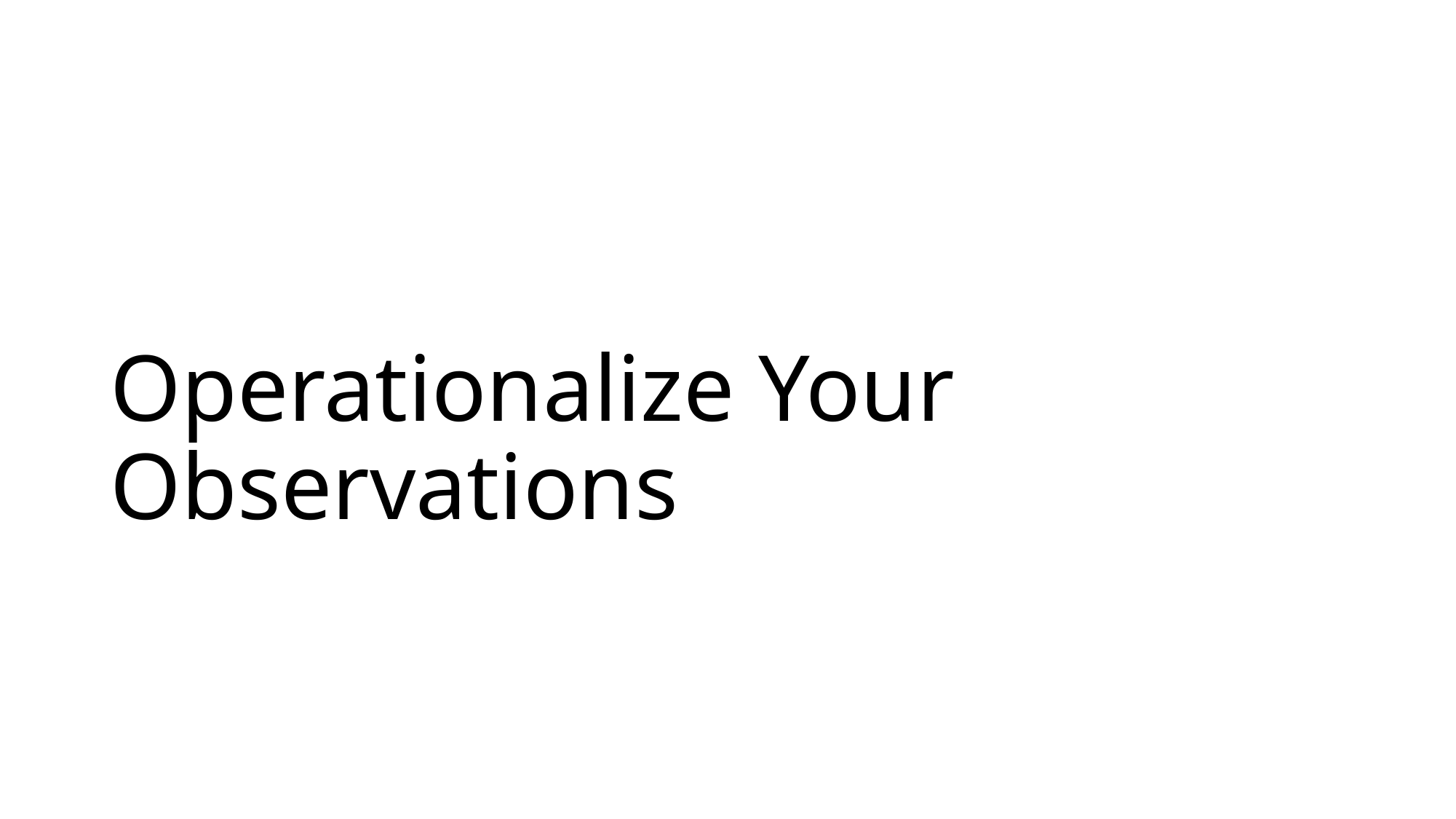

# Operationalize Your Observations

## Slide 14
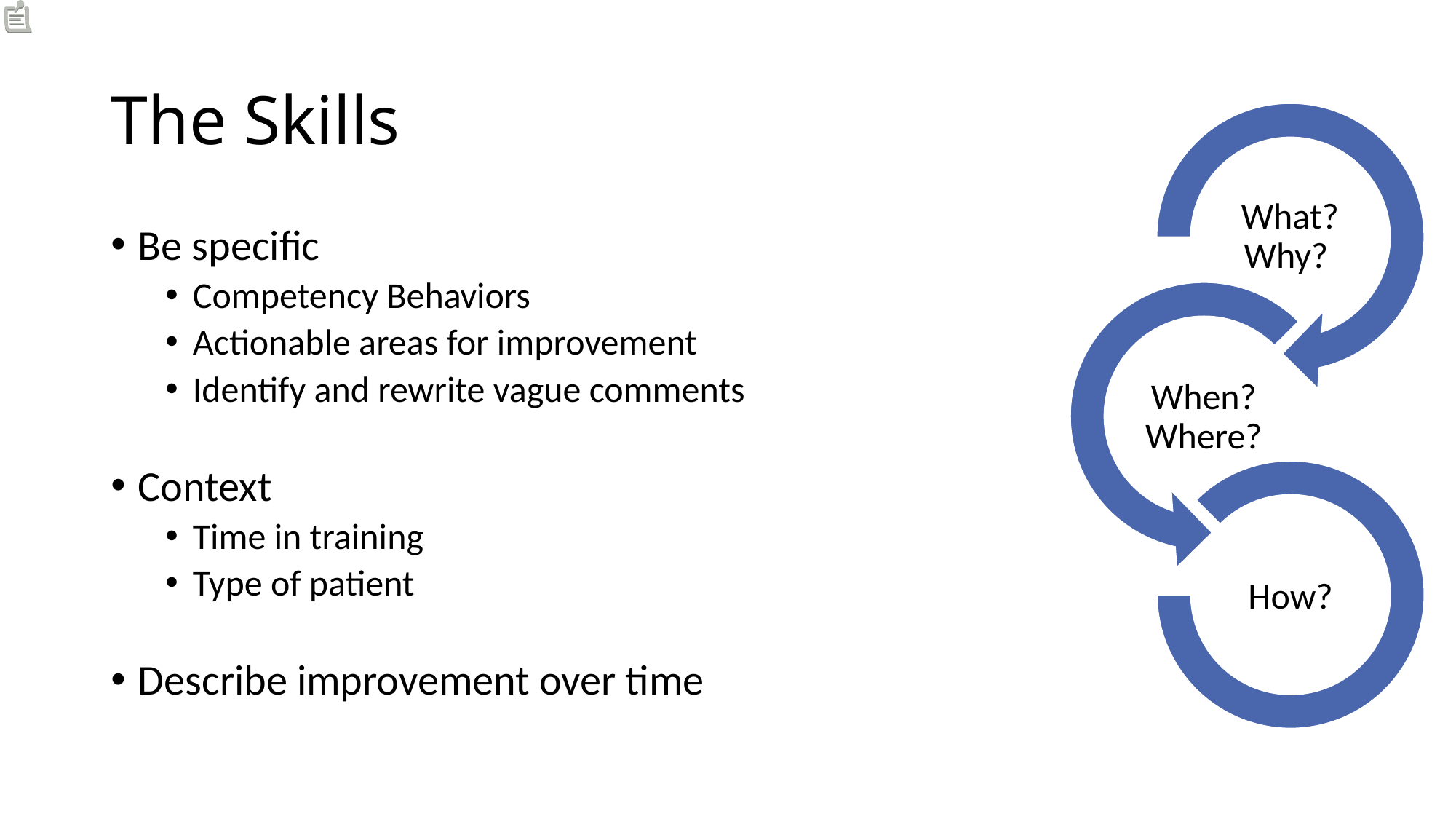

# The Skills
Be specific
Competency Behaviors
Actionable areas for improvement
Identify and rewrite vague comments
Context
Time in training
Type of patient
Describe improvement over time

## Slide 15
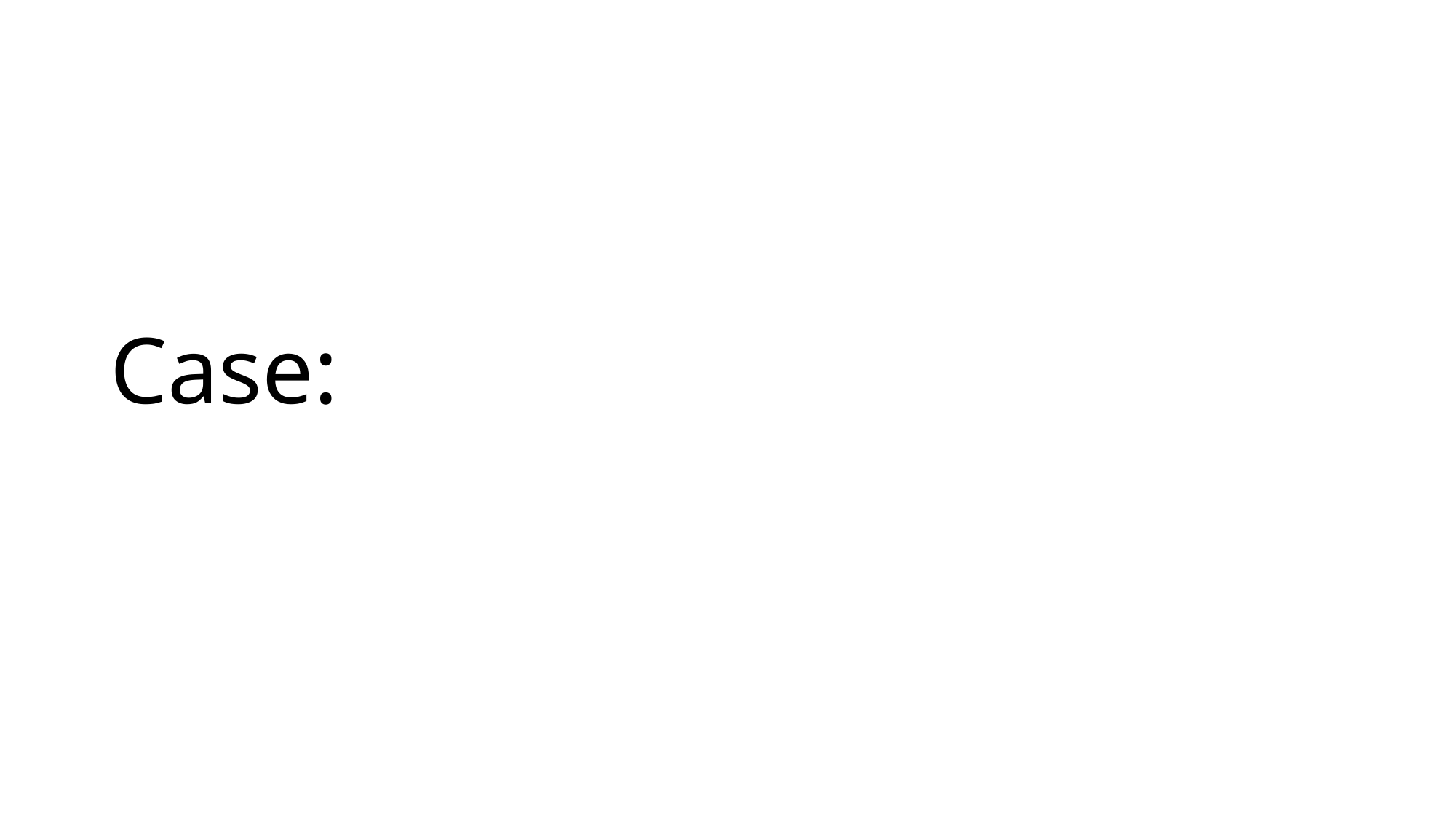

# Case:

## Slide 16
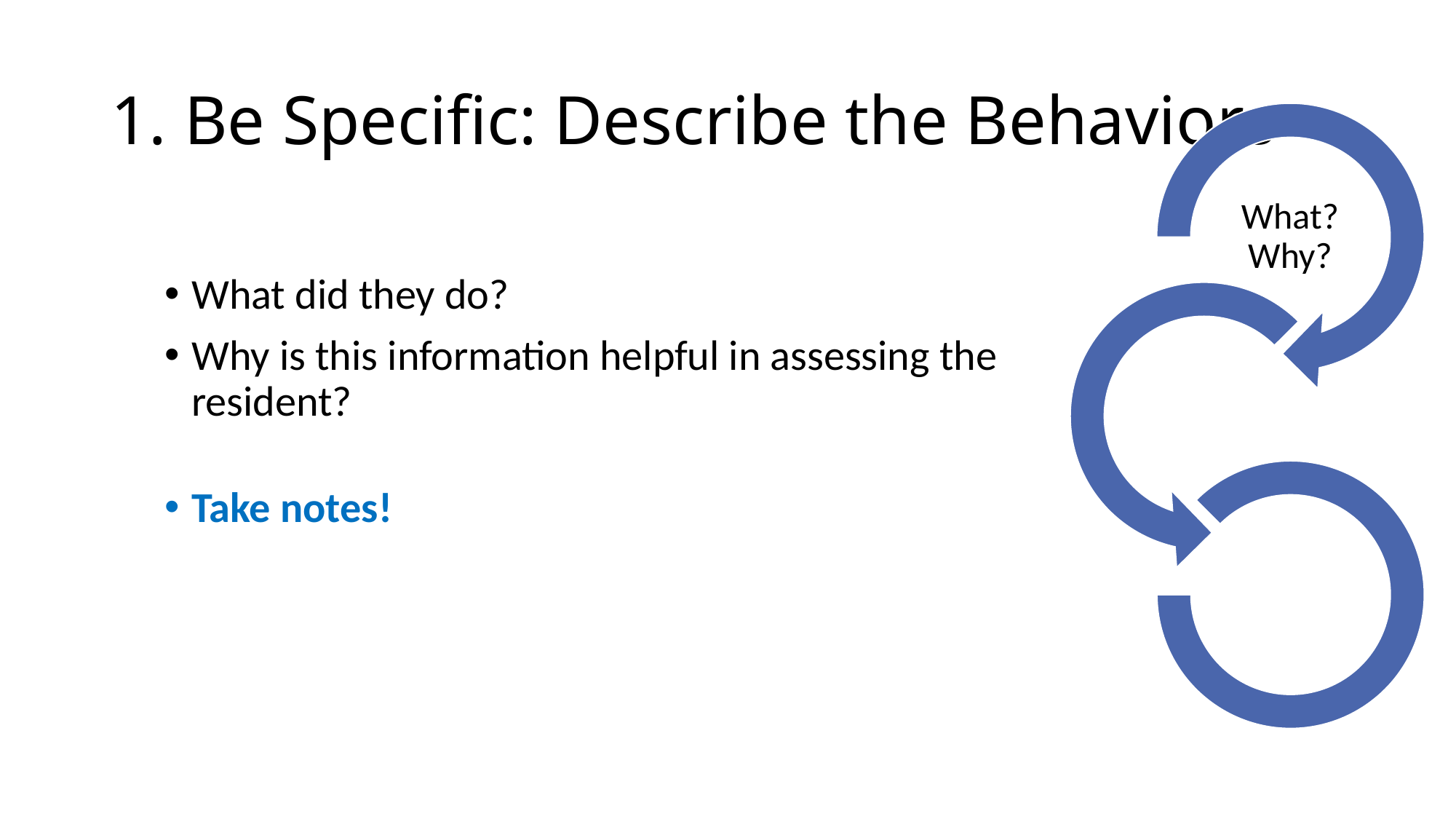

# 1. Be Specific: Describe the Behaviors
What did they do?
Why is this information helpful in assessing the resident?
Take notes!

## Slide 17
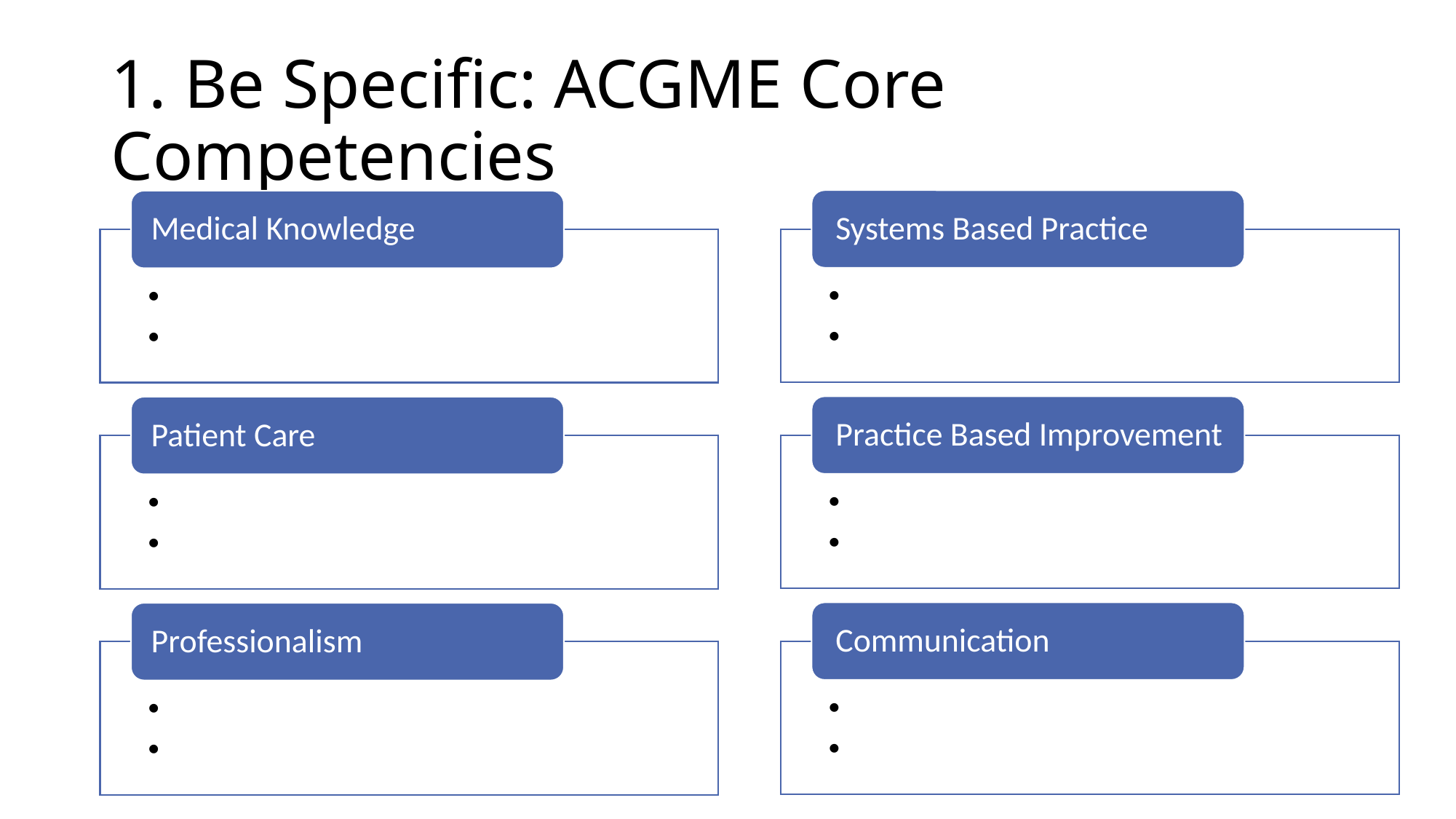

# 1. Be Specific: ACGME Core Competencies
Medical Knowledge
Patient Care
Professionalism

## Slide 18
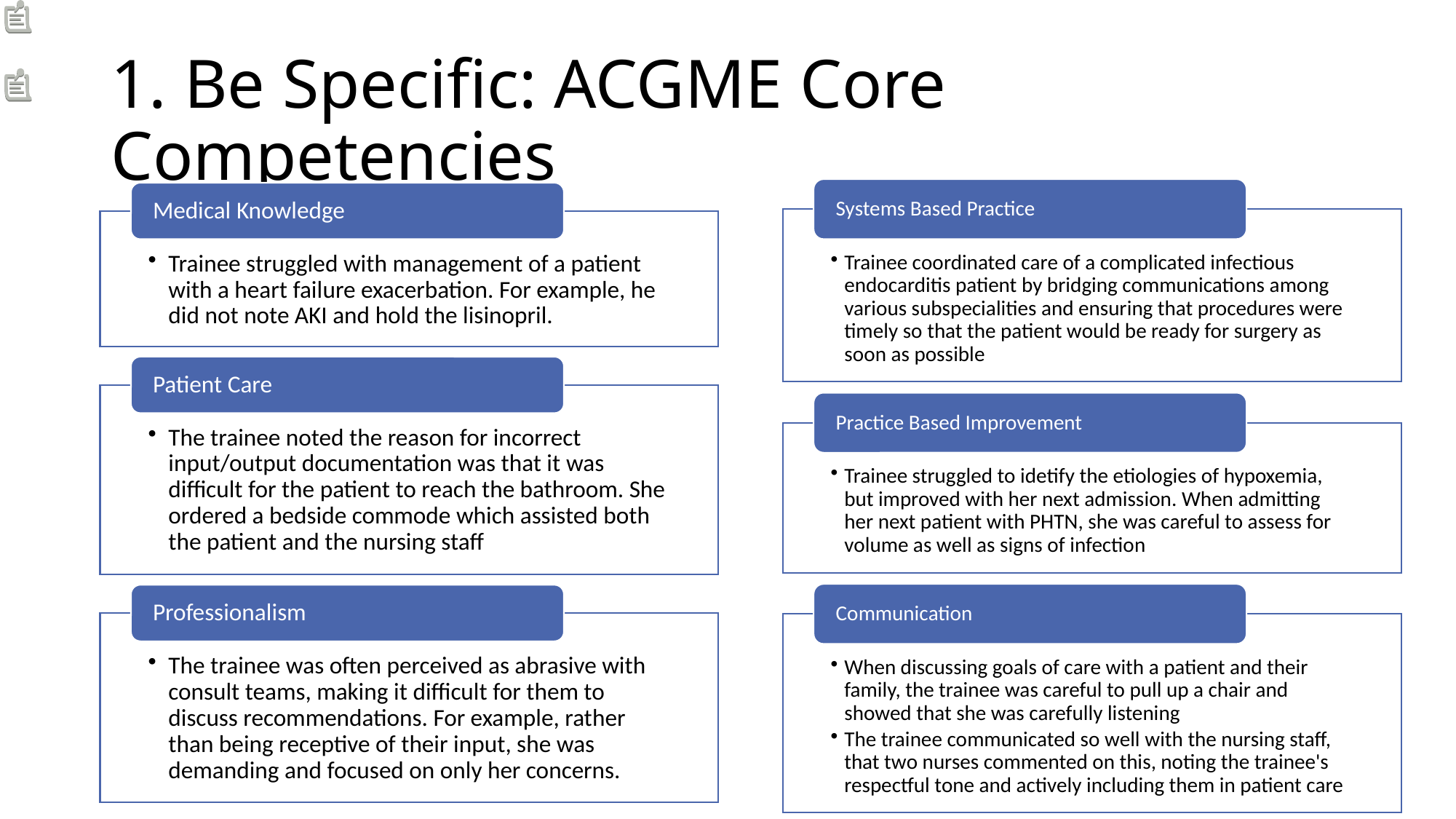

# 1. Be Specific: ACGME Core Competencies

## Slide 19
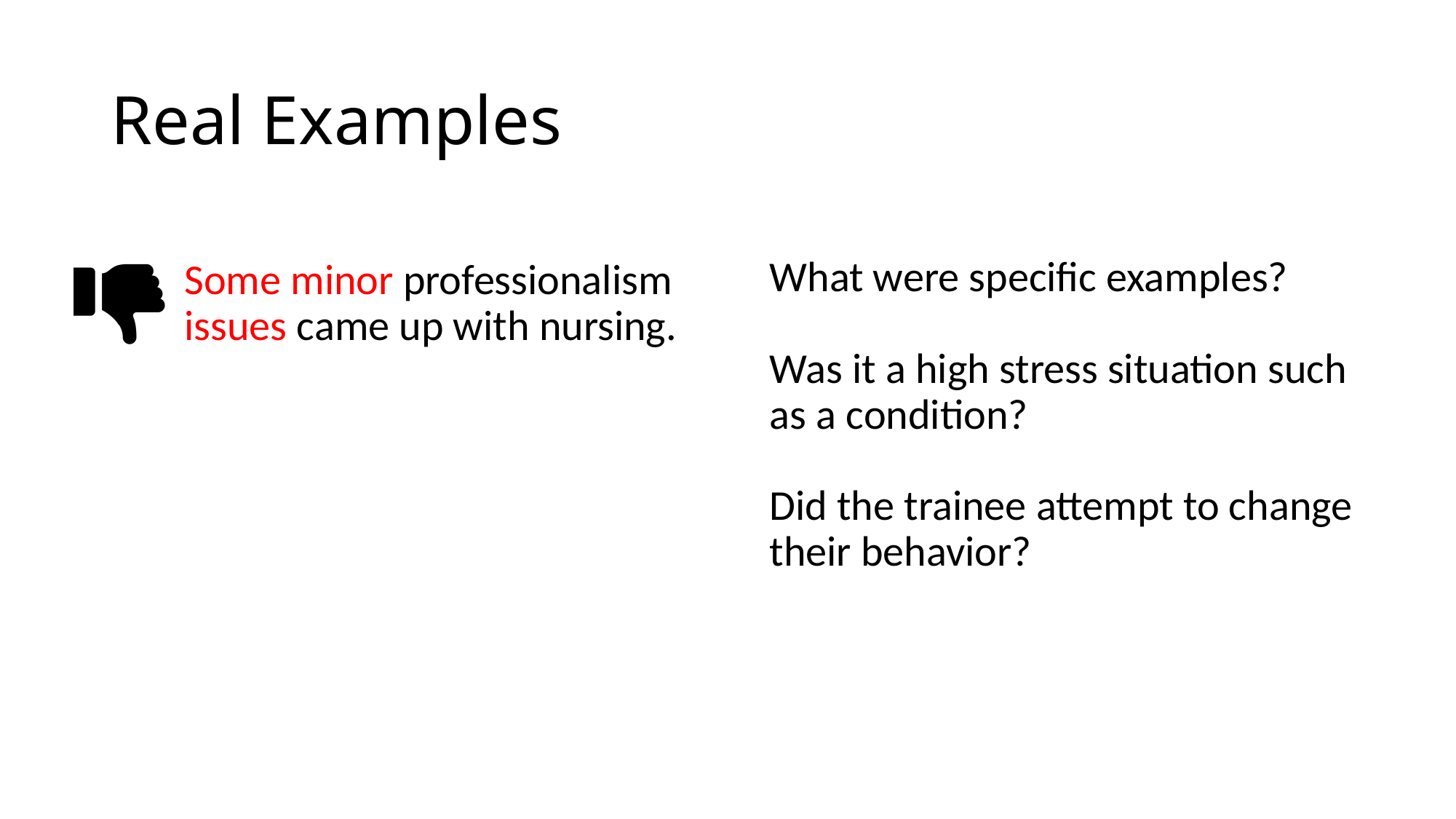

# Real Examples
Some minor professionalism issues came up with nursing.
What were specific examples?
Was it a high stress situation such as a condition?
Did the trainee attempt to change their behavior?

## Slide 20
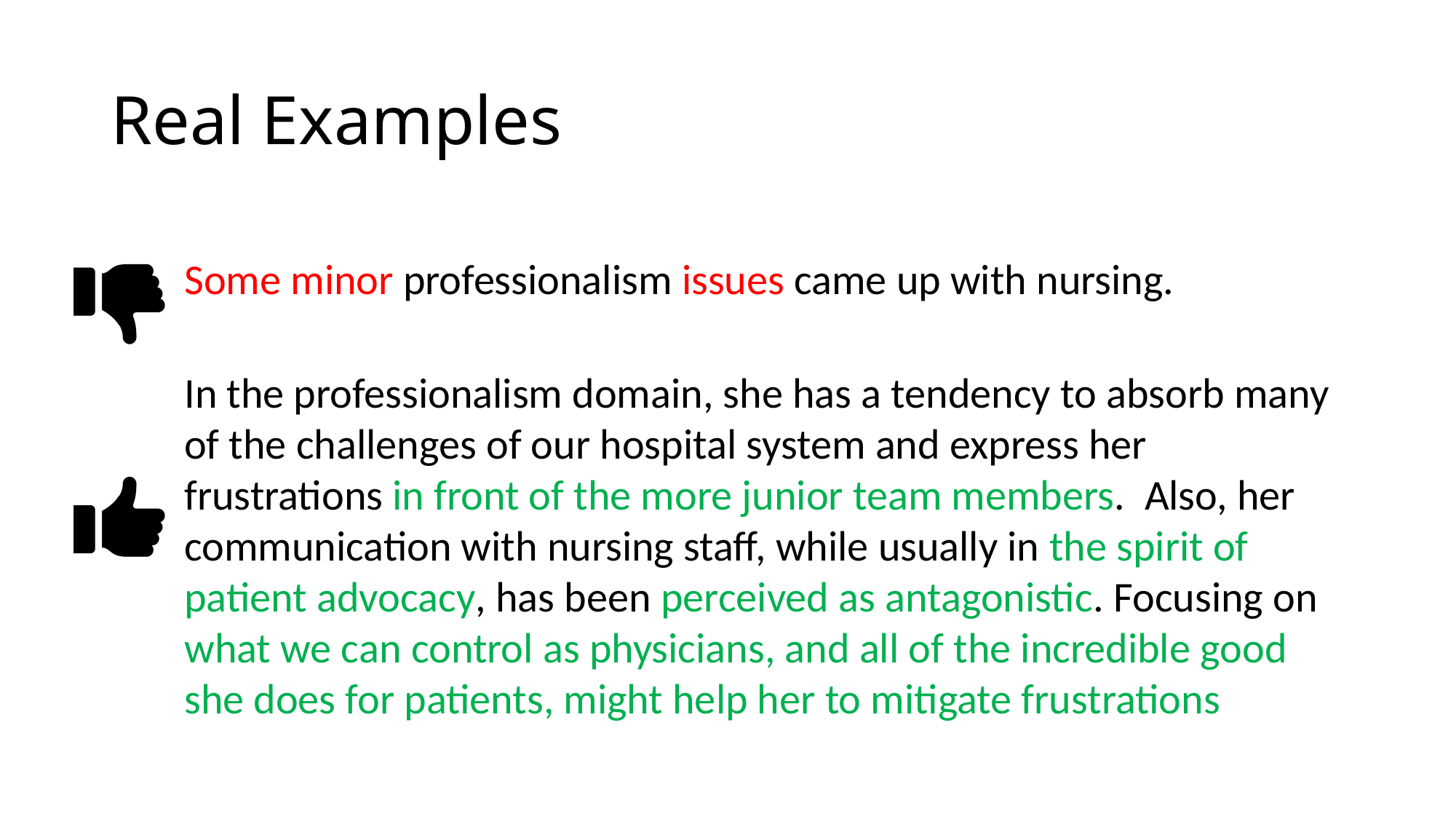

# Real Examples
Some minor professionalism issues came up with nursing.
In the professionalism domain, she has a tendency to absorb many of the challenges of our hospital system and express her frustrations in front of the more junior team members.  Also, her communication with nursing staff, while usually in the spirit of patient advocacy, has been perceived as antagonistic. Focusing on what we can control as physicians, and all of the incredible good she does for patients, might help her to mitigate frustrations

## Slide 21
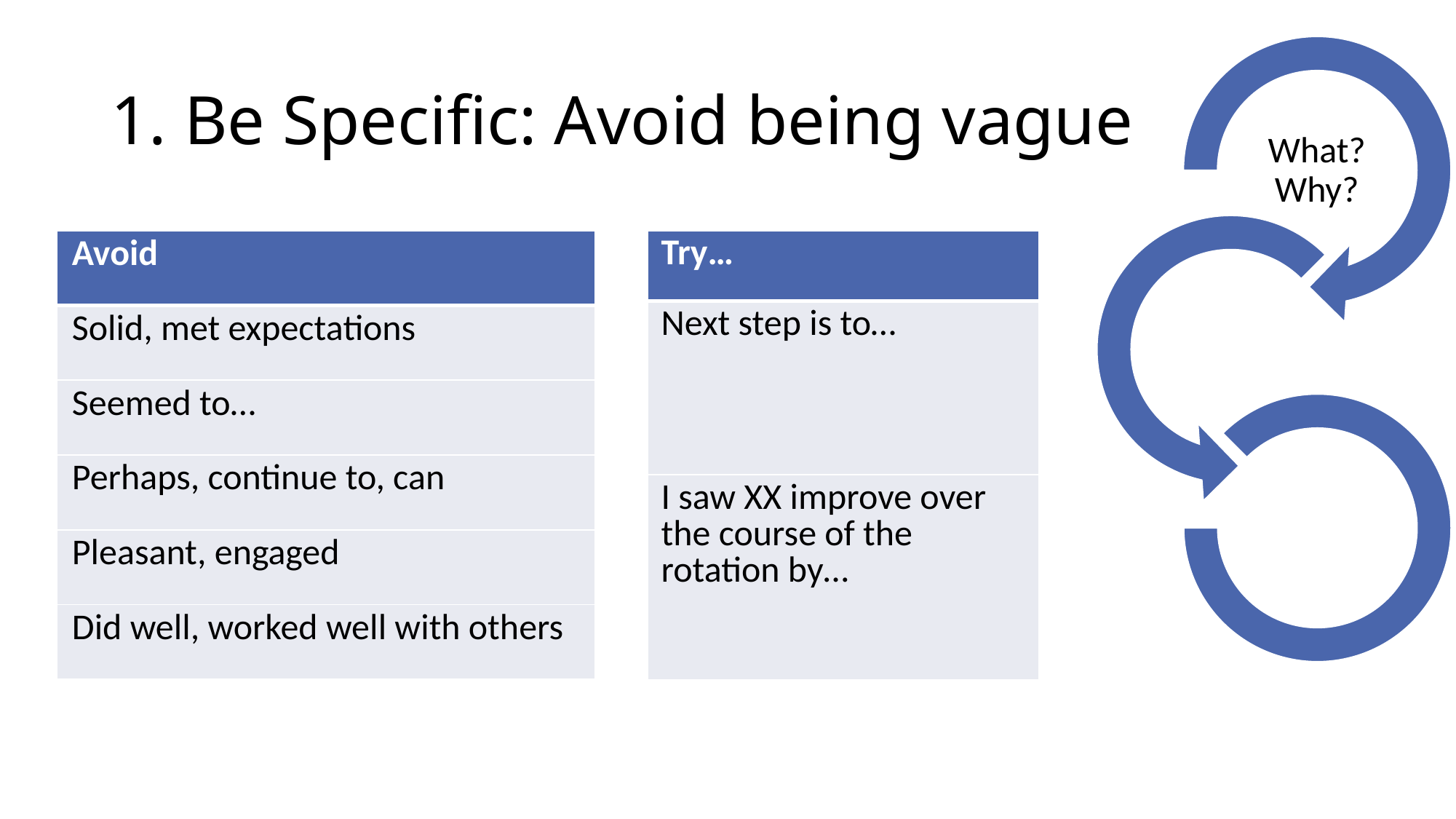

# 1. Be Specific: Avoid being vague
| Avoid |
| --- |
| Solid, met expectations |
| Seemed to… |
| Perhaps, continue to, can |
| Pleasant, engaged |
| Did well, worked well with others |
| Try… |
| --- |
| Next step is to… |
| I saw XX improve over the course of the rotation by… |

## Slide 22
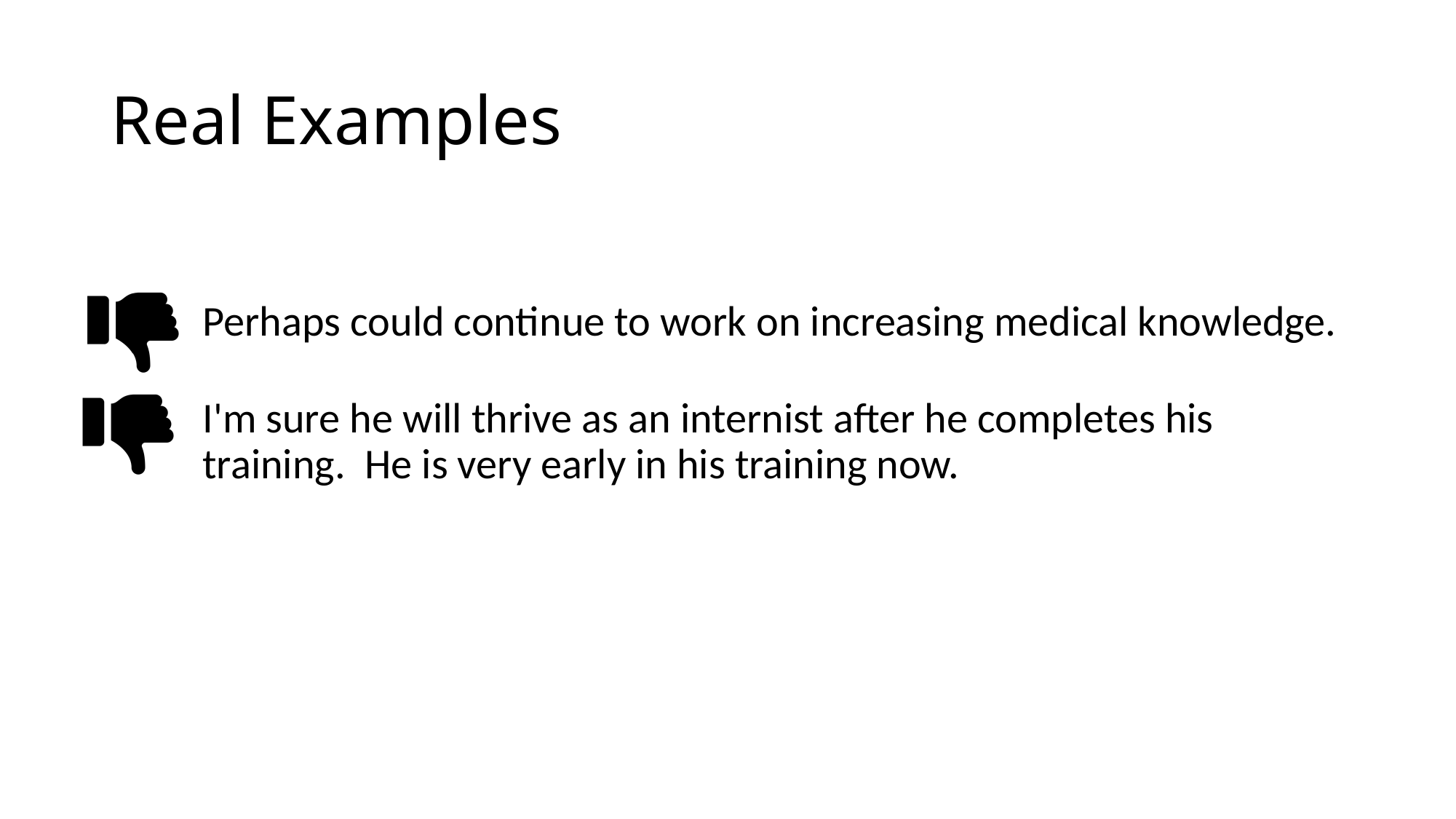

# Real Examples
Perhaps could continue to work on increasing medical knowledge.
I'm sure he will thrive as an internist after he completes his training.  He is very early in his training now.

## Slide 23
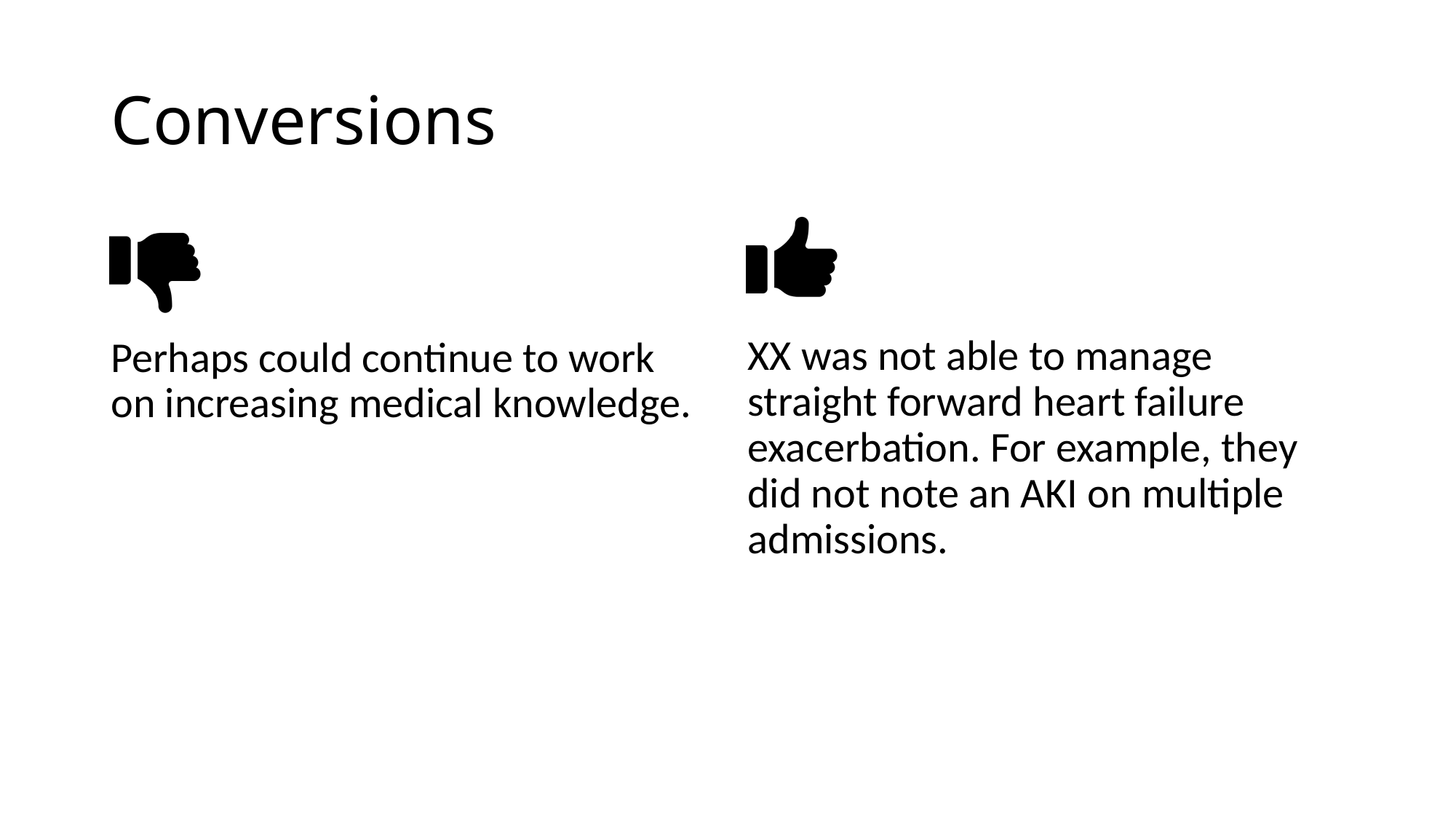

# Conversions
Perhaps could continue to work on increasing medical knowledge.
XX was not able to manage straight forward heart failure exacerbation. For example, they did not note an AKI on multiple admissions.

## Slide 24
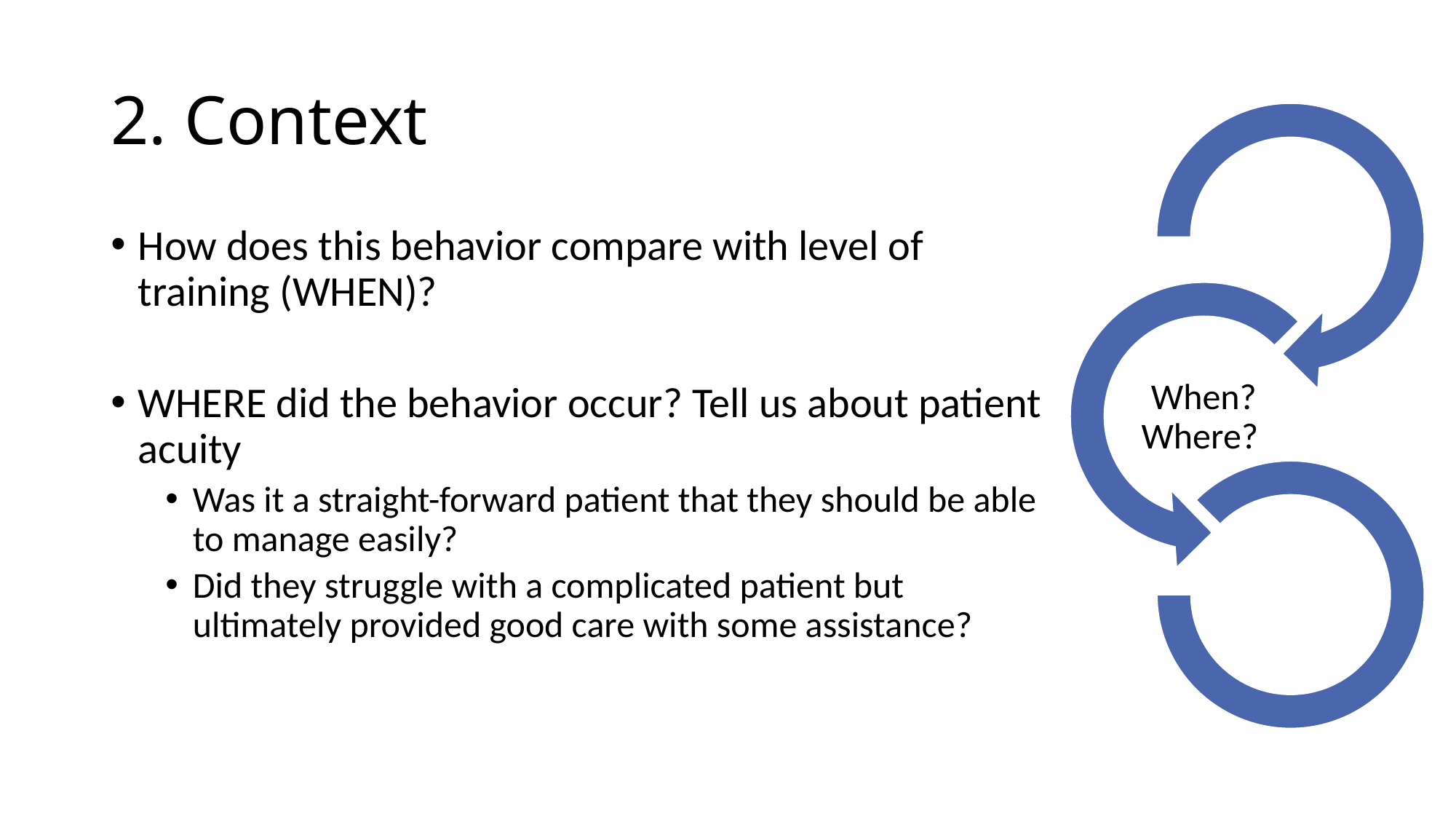

# 2. Context
How does this behavior compare with level of training (WHEN)?
WHERE did the behavior occur? Tell us about patient acuity
Was it a straight-forward patient that they should be able to manage easily?
Did they struggle with a complicated patient but ultimately provided good care with some assistance?

## Slide 25
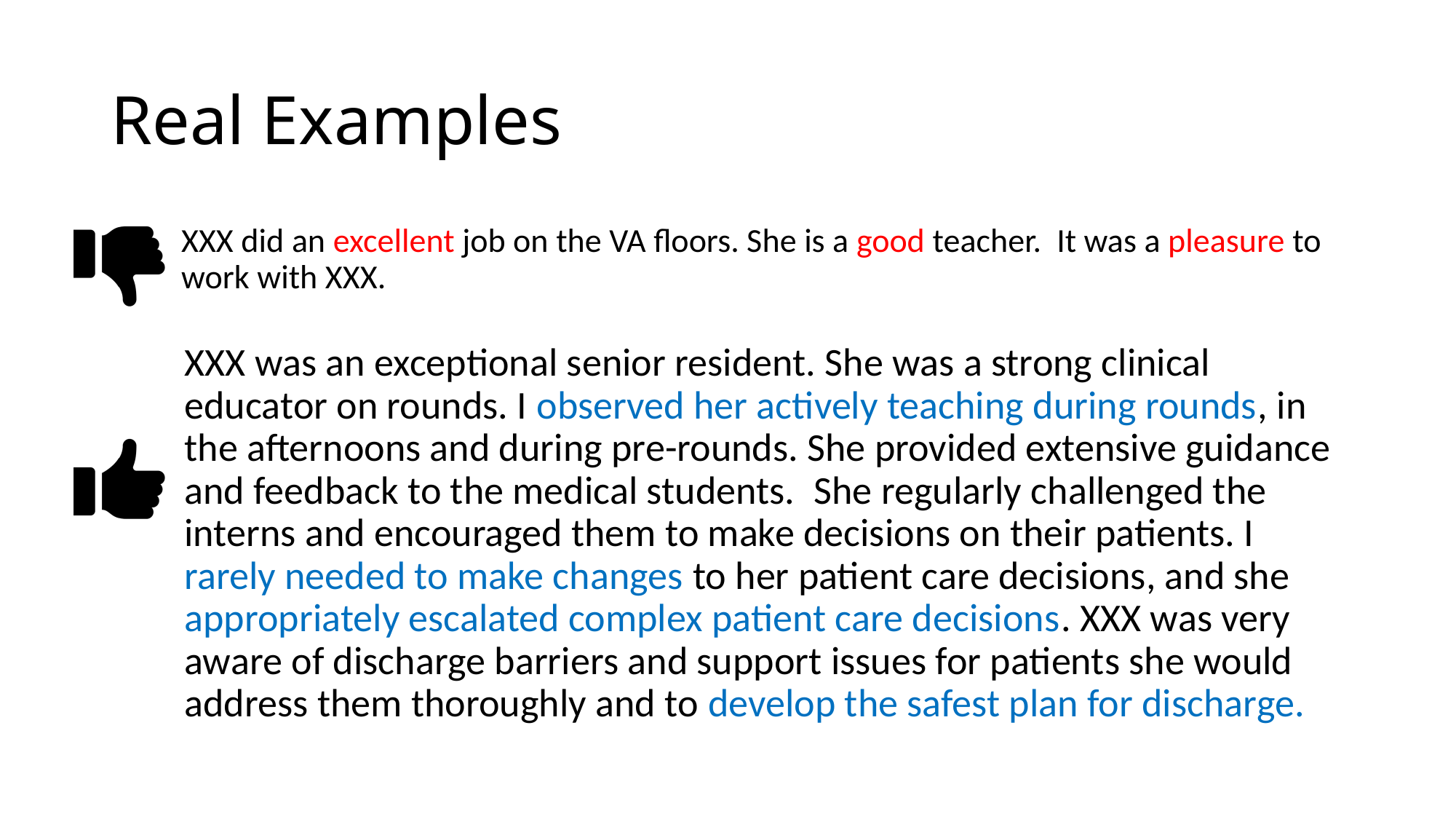

# Real Examples
XXX did an excellent job on the VA floors. She is a good teacher.  It was a pleasure to work with XXX.
XXX was an exceptional senior resident. She was a strong clinical educator on rounds. I observed her actively teaching during rounds, in the afternoons and during pre-rounds. She provided extensive guidance and feedback to the medical students.  She regularly challenged the interns and encouraged them to make decisions on their patients. I rarely needed to make changes to her patient care decisions, and she appropriately escalated complex patient care decisions. XXX was very aware of discharge barriers and support issues for patients she would address them thoroughly and to develop the safest plan for discharge.

## Slide 26
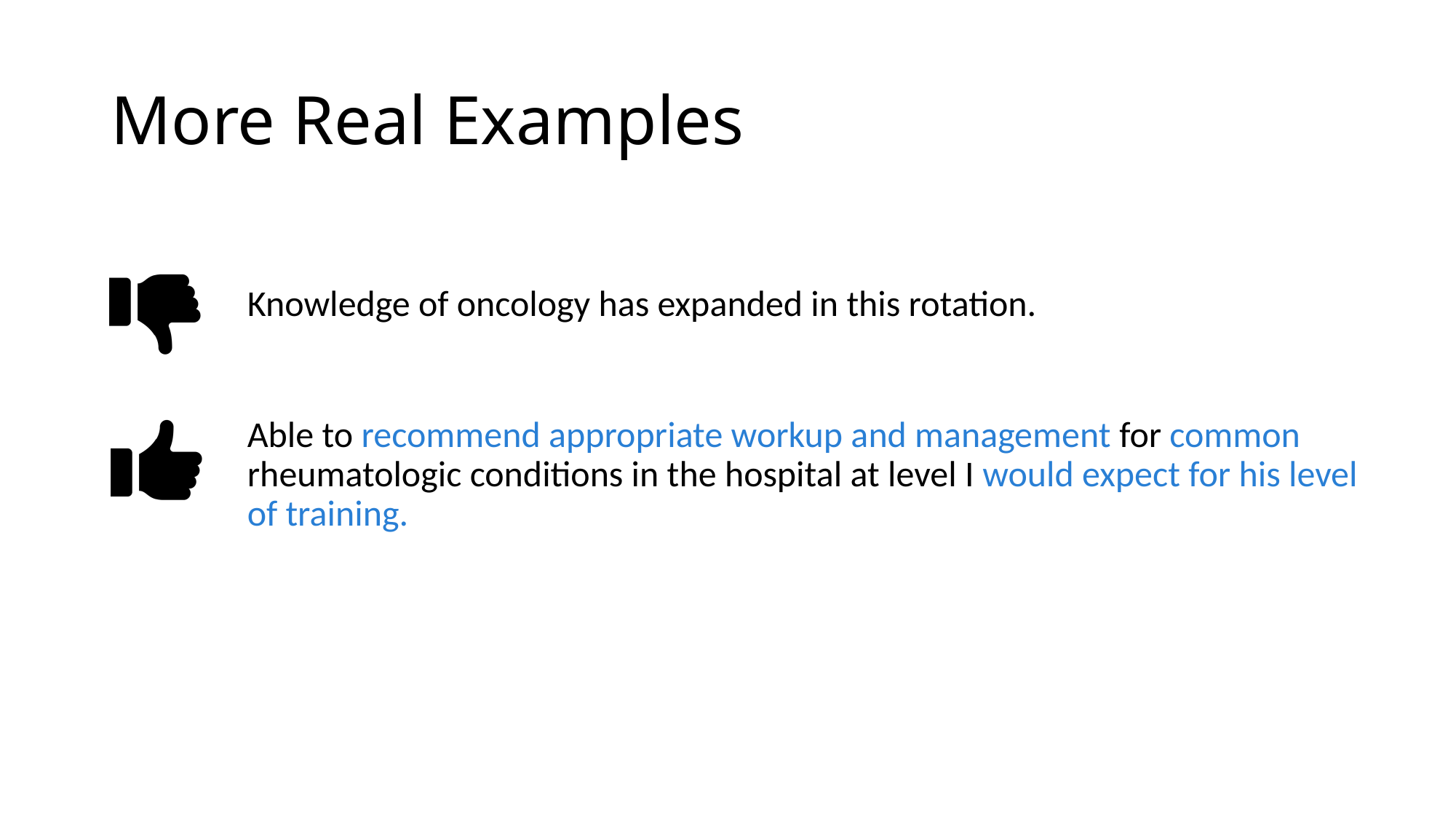

# More Real Examples
Knowledge of oncology has expanded in this rotation.
Able to recommend appropriate workup and management for common rheumatologic conditions in the hospital at level I would expect for his level of training.

## Slide 27
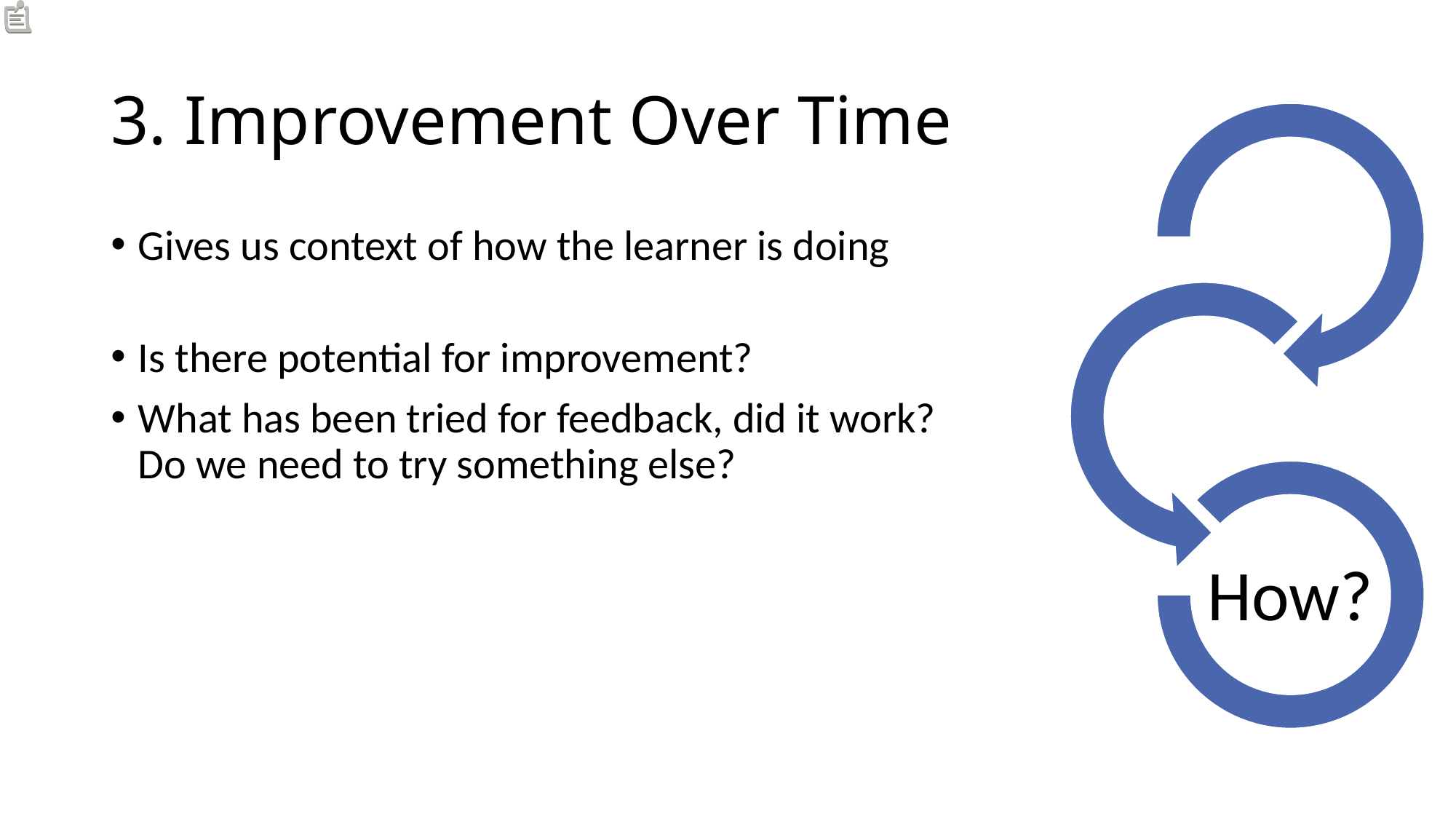

# 3. Improvement Over Time
Gives us context of how the learner is doing
Is there potential for improvement?
What has been tried for feedback, did it work?
Do we need to try something else?

## Slide 28
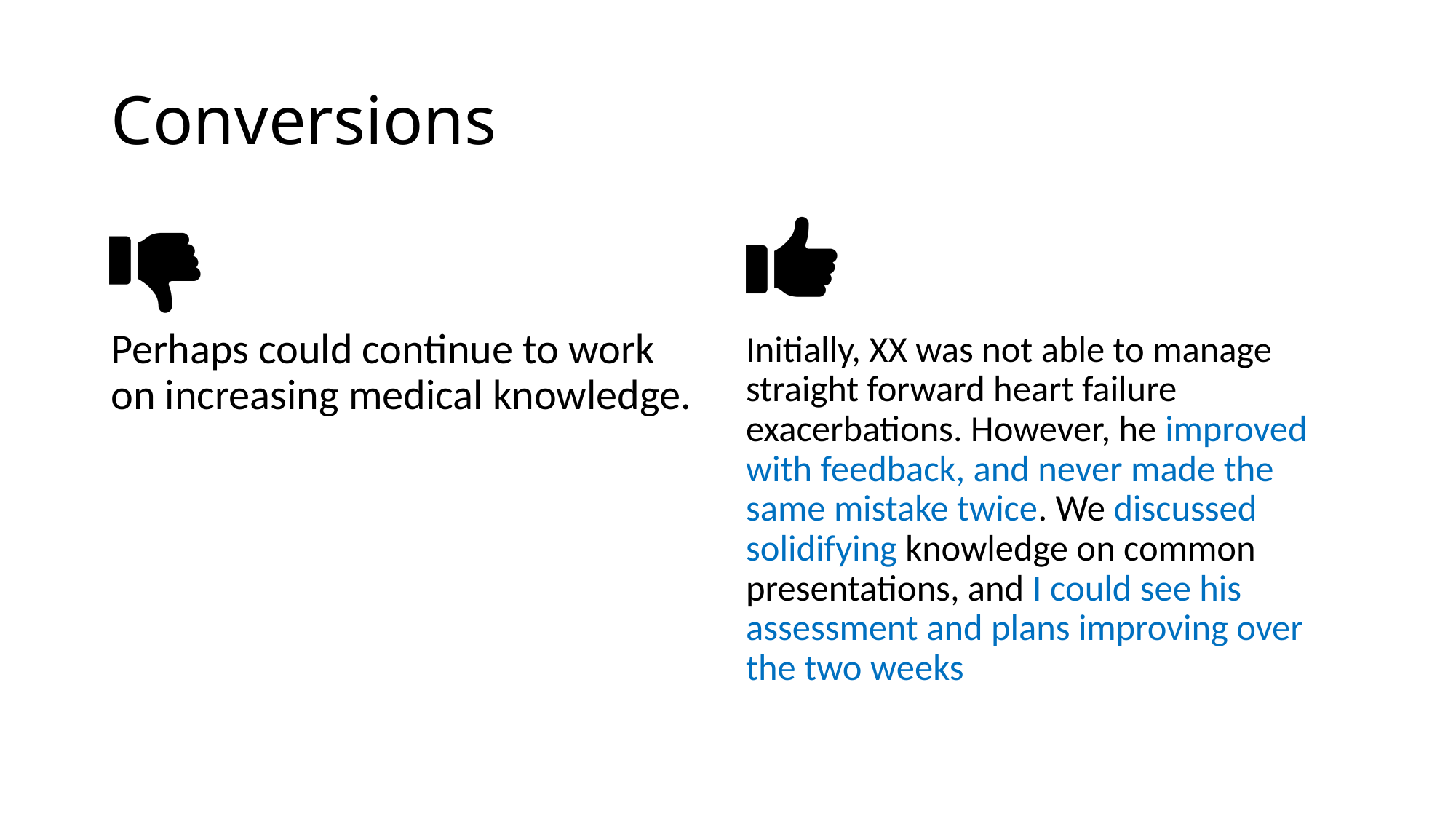

# Conversions
Initially, XX was not able to manage straight forward heart failure exacerbations. However, he improved with feedback, and never made the same mistake twice. We discussed solidifying knowledge on common presentations, and I could see his assessment and plans improving over the two weeks
Perhaps could continue to work on increasing medical knowledge.

## Slide 29
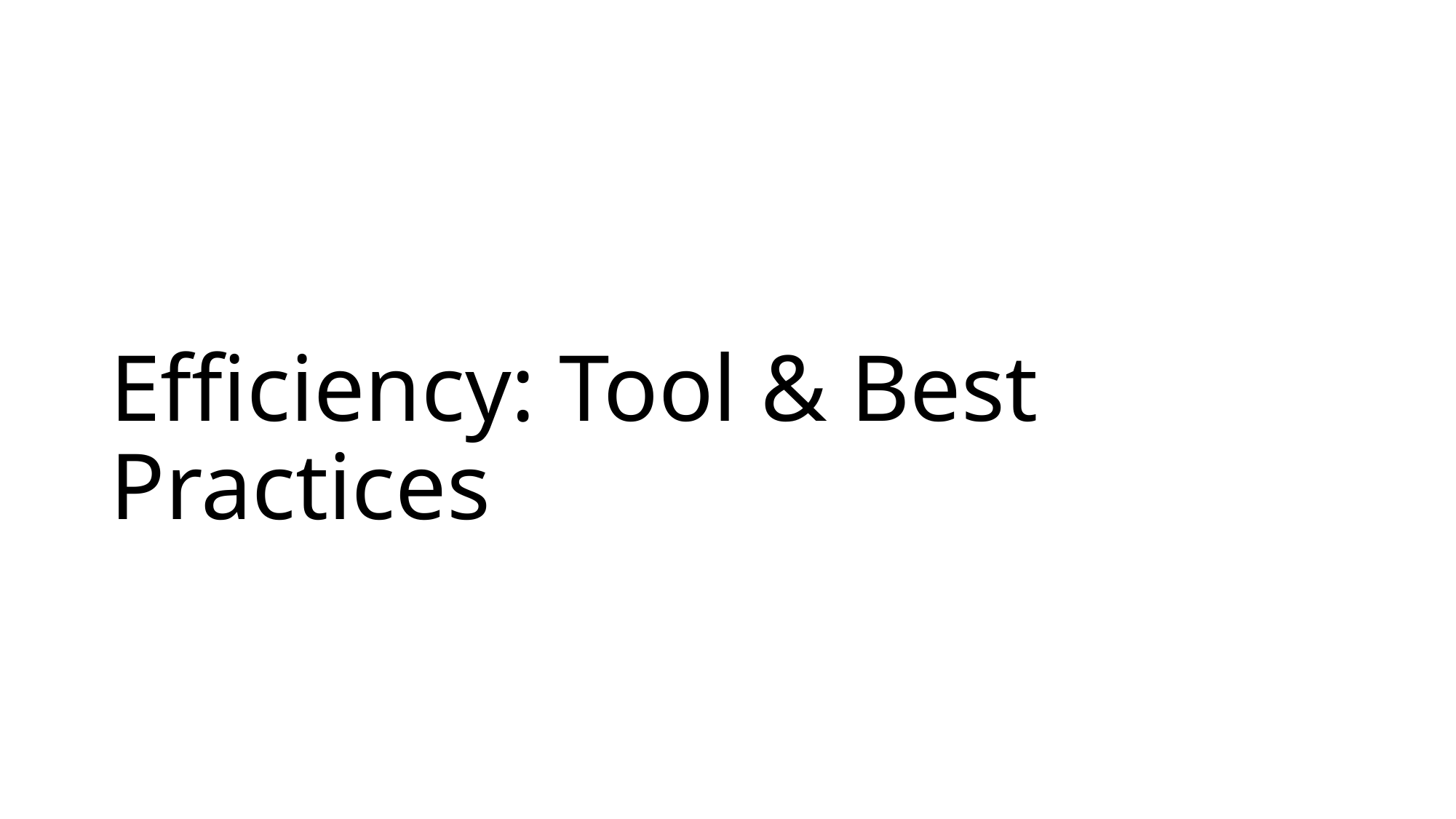

# Efficiency: Tool & Best Practices

## Slide 30
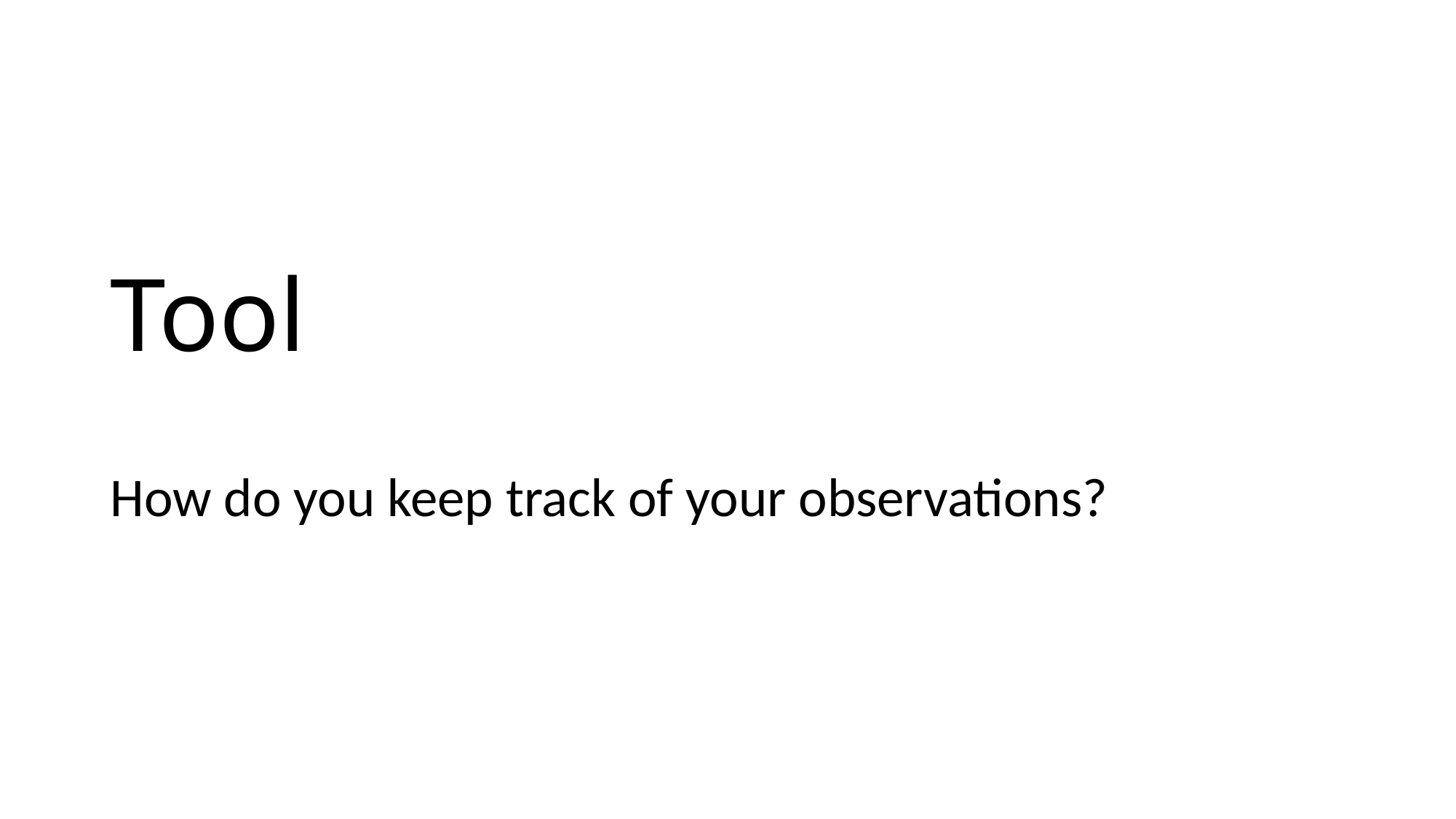

# Tool
How do you keep track of your observations?

## Slide 31
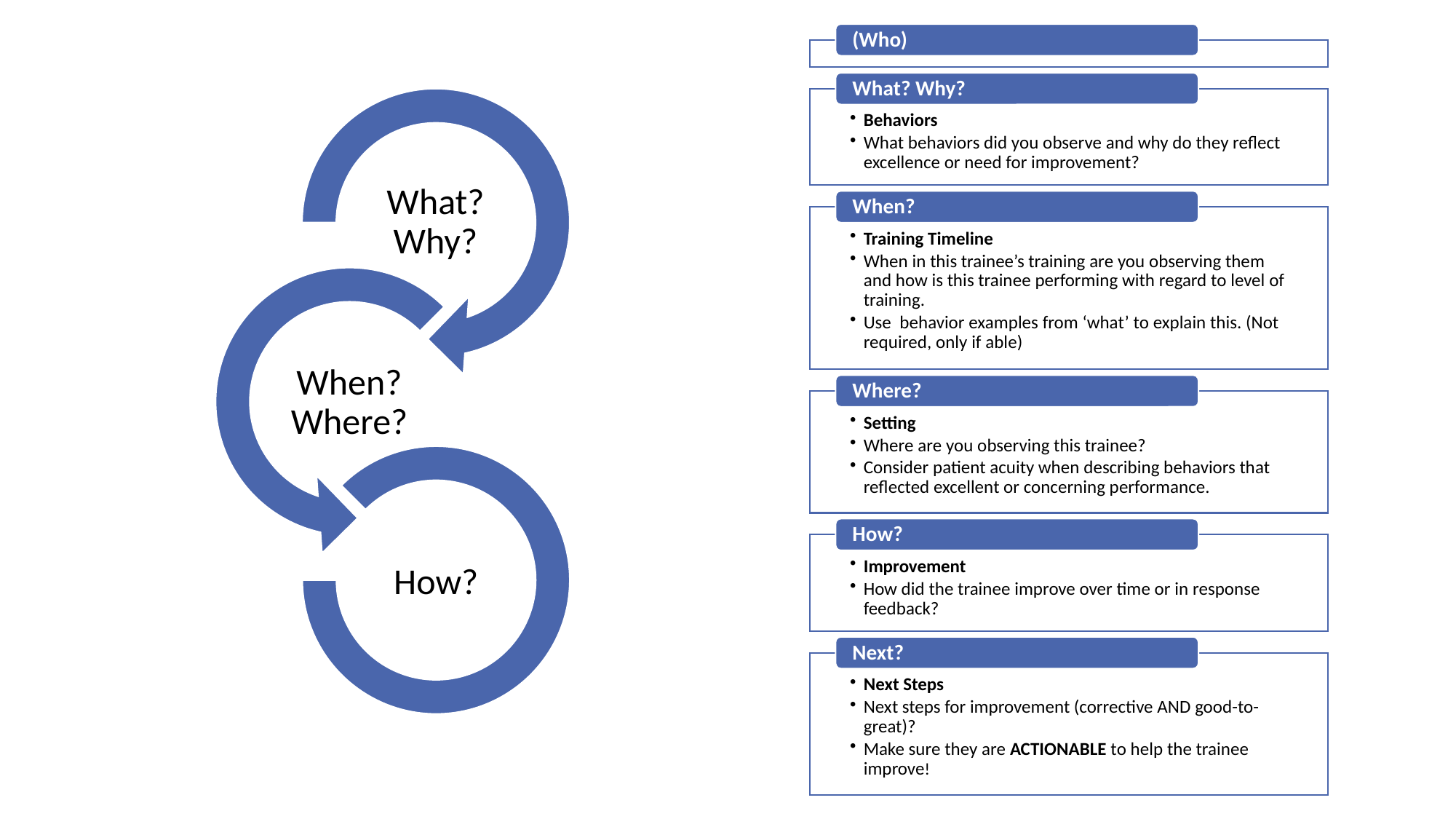

# Practice!
Let’s go back to the video. With this new knowledge, please rewrite your evaluation of the learner

## Slide 32
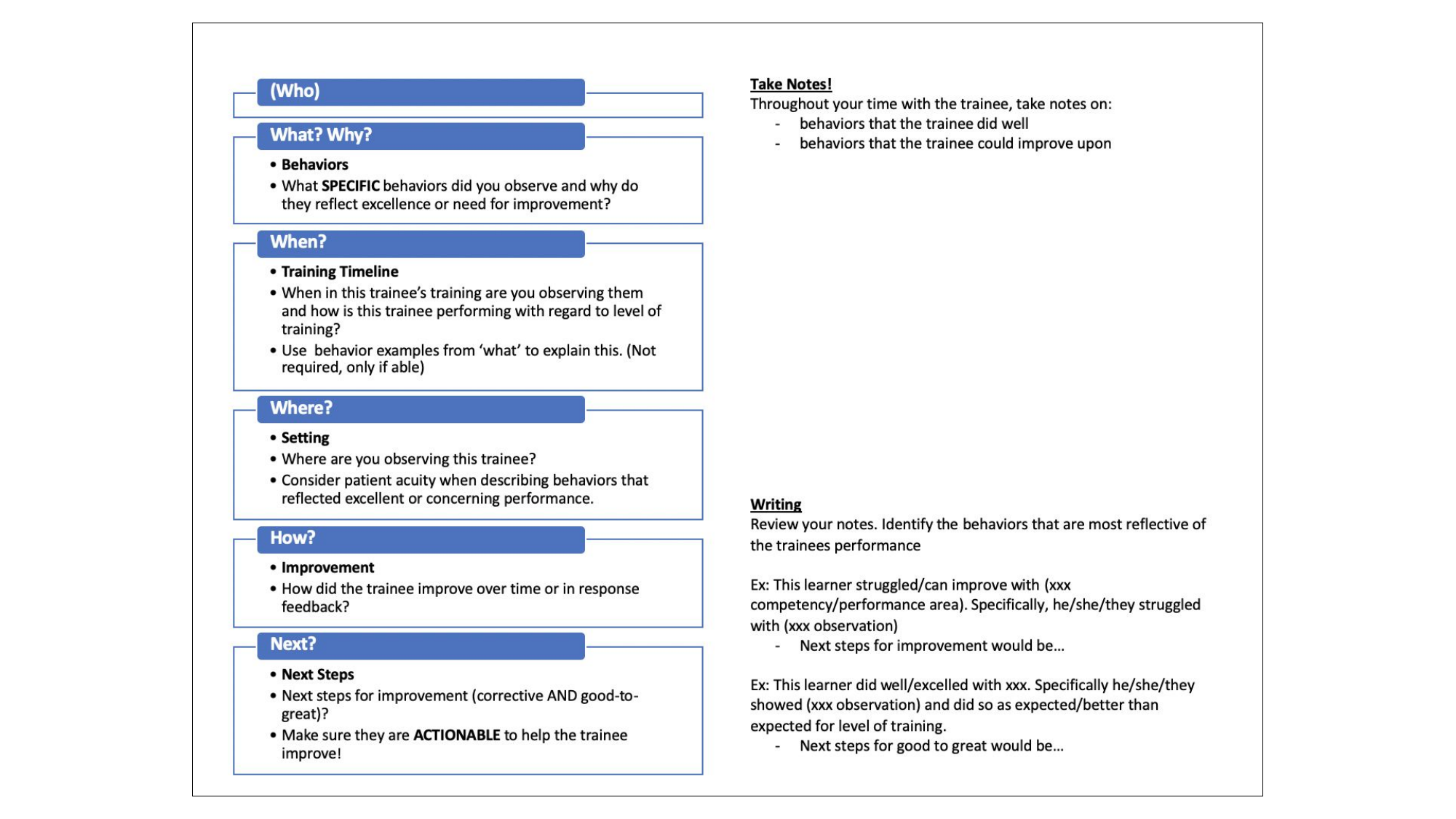

## Slide 33
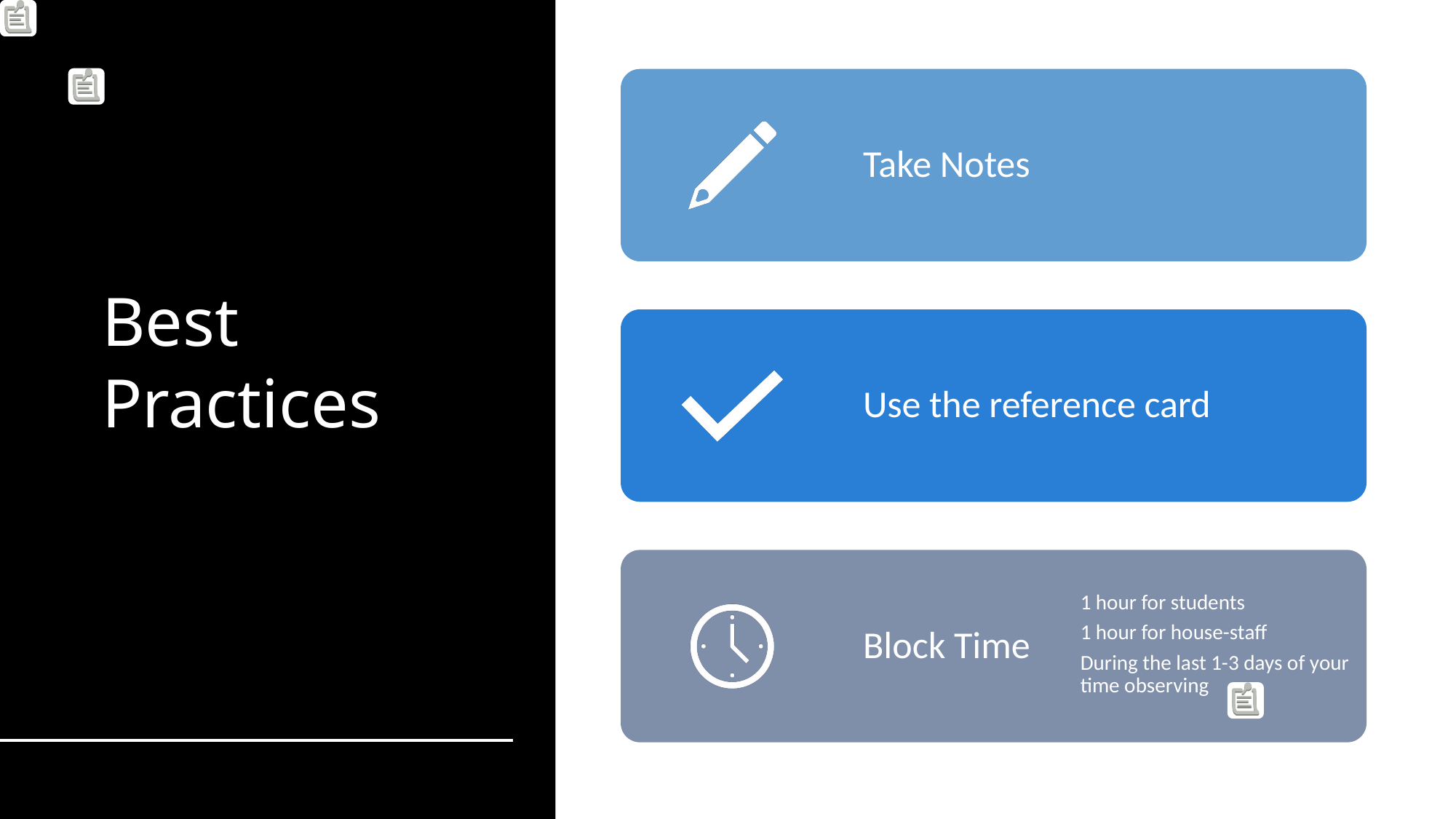

# Best Practices
Take Notes
Use the reference card
1 hour for students
1 hour for house-staff
During the last 1-3 days of your time observing
Block Time

## Slide 34
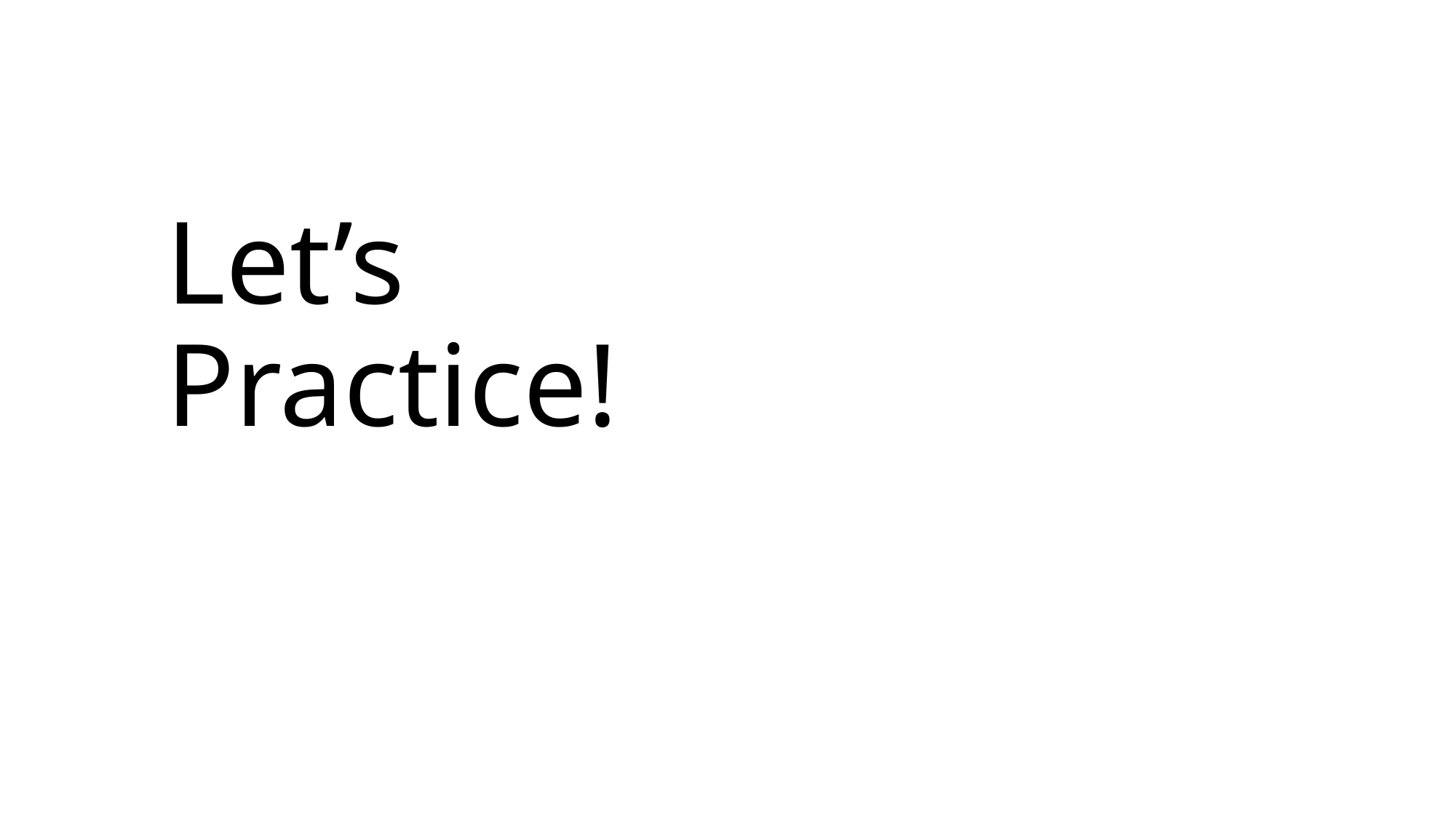

# Let’s Practice!
Small Group Instructions:
Learner Video (5 min)
Practice Writing (2 min)
Discussion (10 min)

## Slide 35
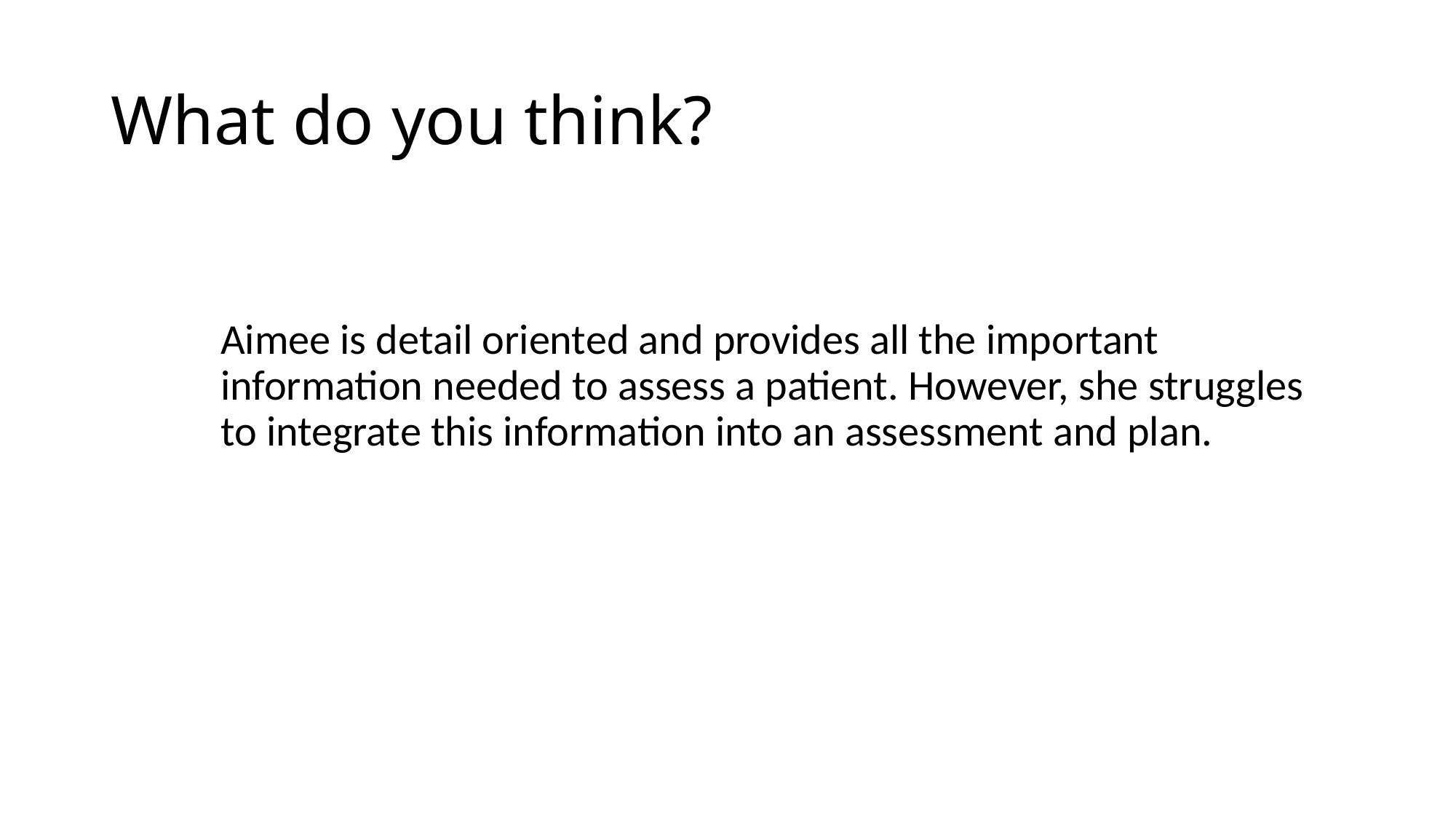

# What do you think?
Aimee is detail oriented and provides all the important information needed to assess a patient. However, she struggles to integrate this information into an assessment and plan.

## Slide 36
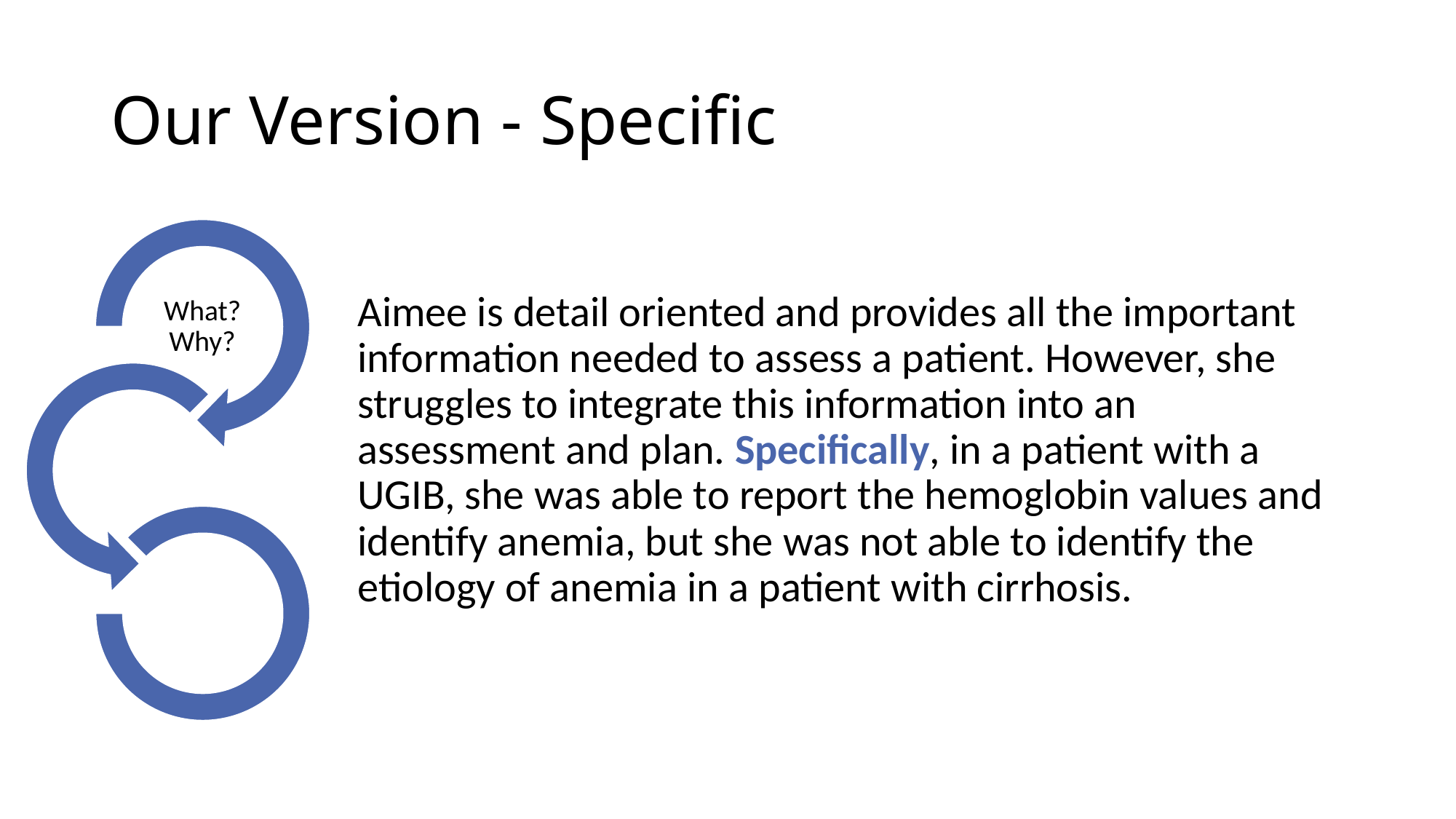

# Our Version - Specific
Aimee is detail oriented and provides all the important information needed to assess a patient. However, she struggles to integrate this information into an assessment and plan. Specifically, in a patient with a UGIB, she was able to report the hemoglobin values and identify anemia, but she was not able to identify the etiology of anemia in a patient with cirrhosis.

## Slide 37
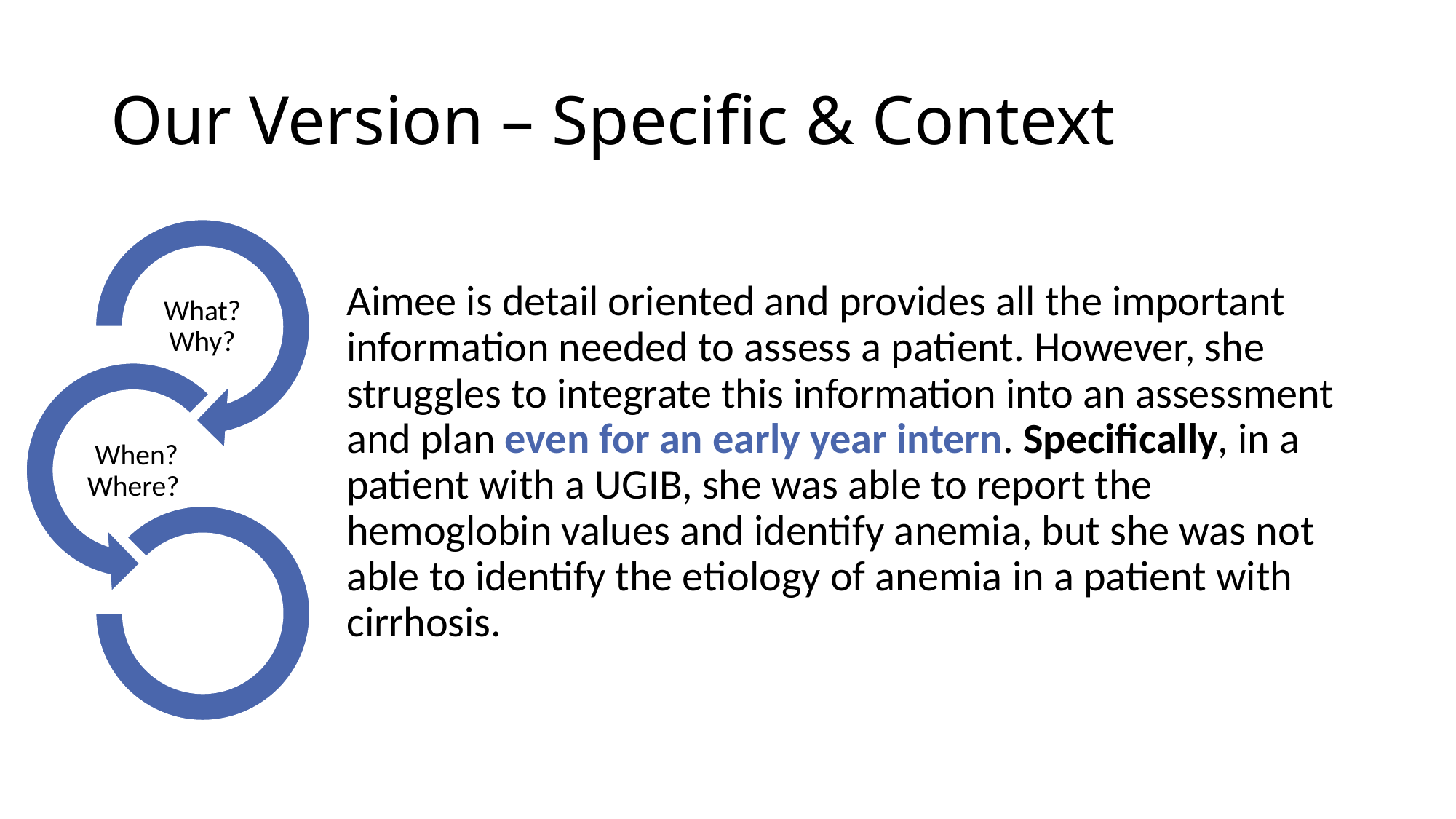

# Our Version – Specific & Context
Aimee is detail oriented and provides all the important information needed to assess a patient. However, she struggles to integrate this information into an assessment and plan even for an early year intern. Specifically, in a patient with a UGIB, she was able to report the hemoglobin values and identify anemia, but she was not able to identify the etiology of anemia in a patient with cirrhosis.

## Slide 38
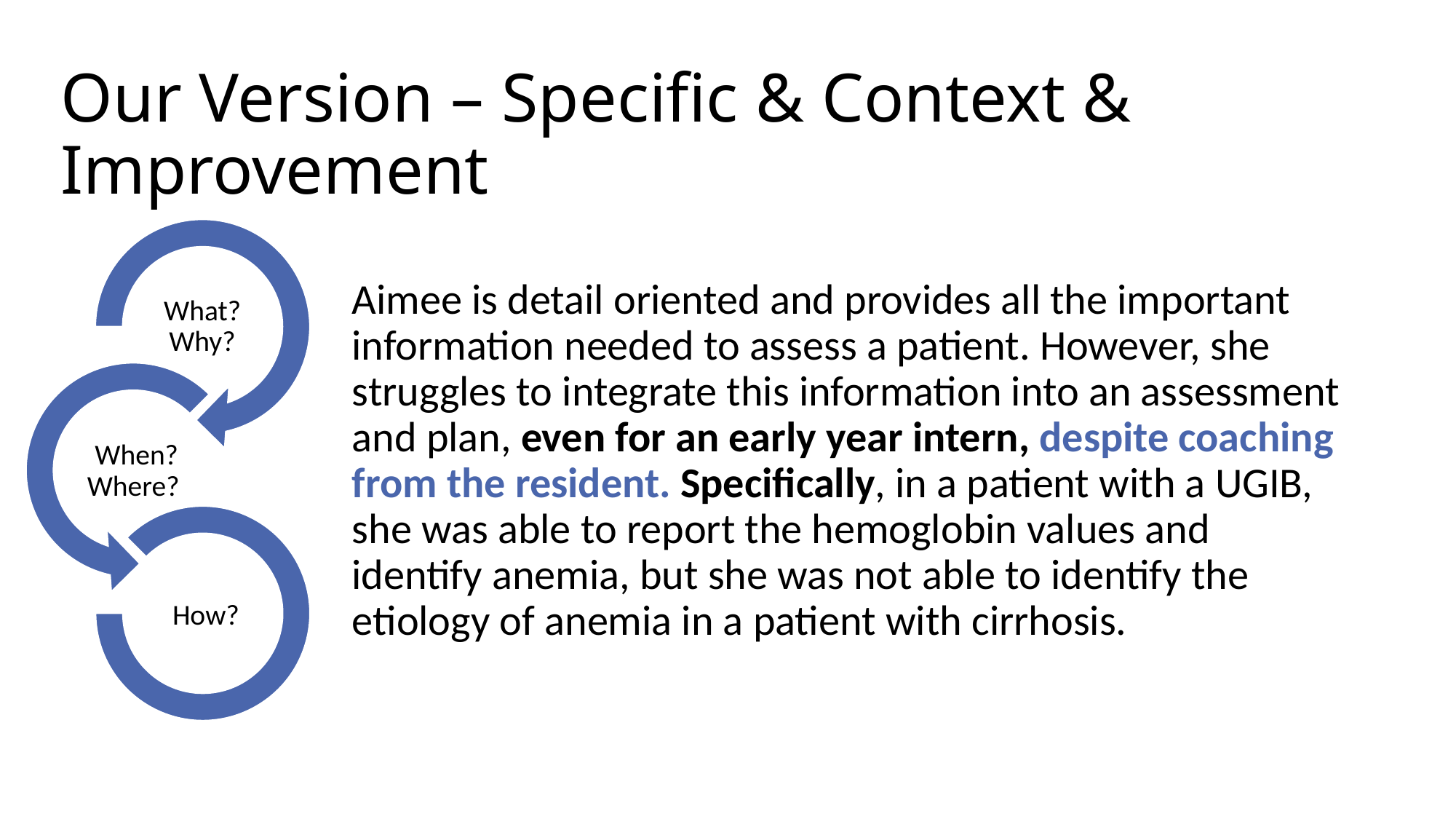

# Our Version – Specific & Context & Improvement
Aimee is detail oriented and provides all the important information needed to assess a patient. However, she struggles to integrate this information into an assessment and plan, even for an early year intern, despite coaching from the resident. Specifically, in a patient with a UGIB, she was able to report the hemoglobin values and identify anemia, but she was not able to identify the etiology of anemia in a patient with cirrhosis.

## Slide 39
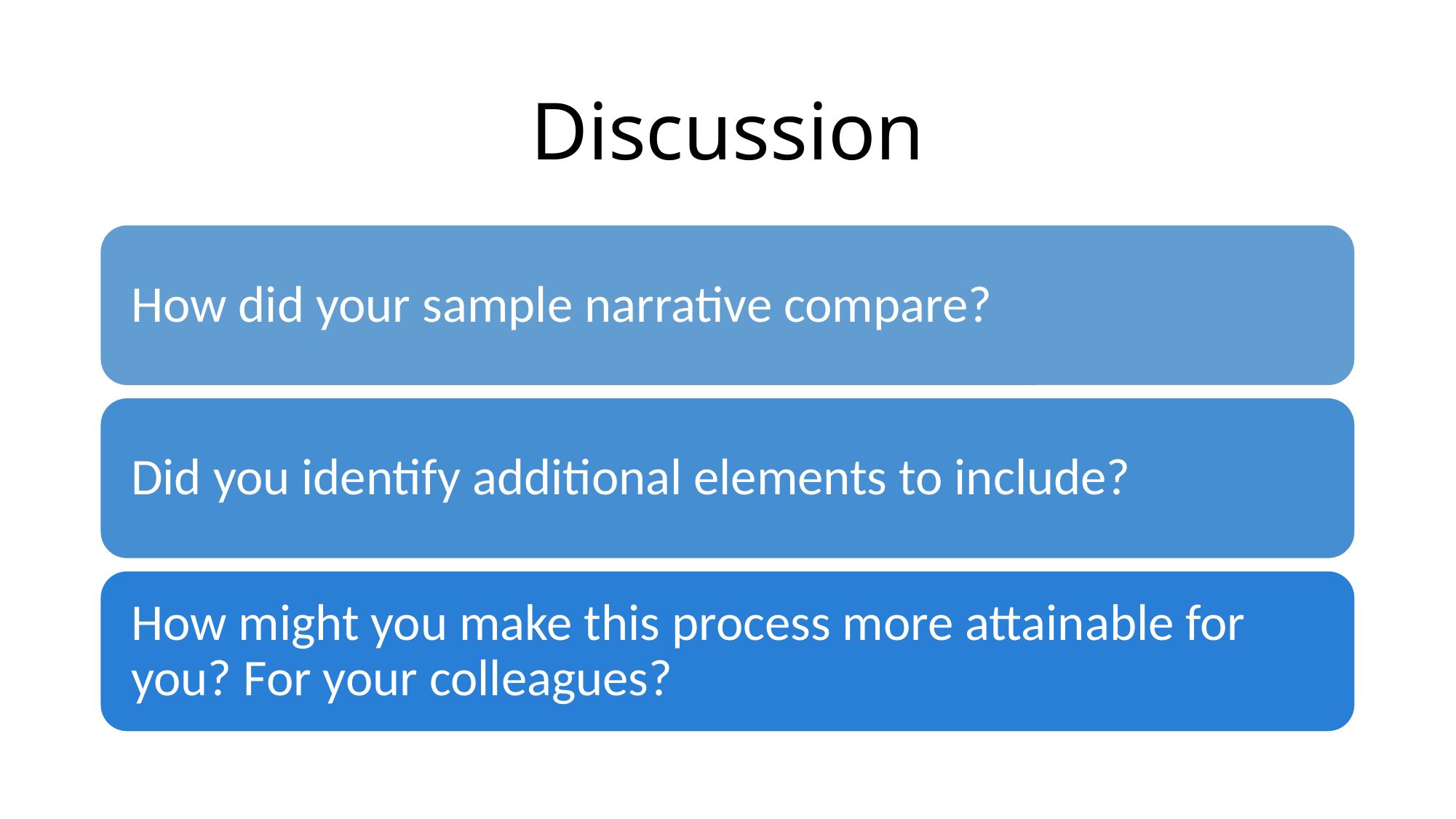

# Discussion

## Slide 40
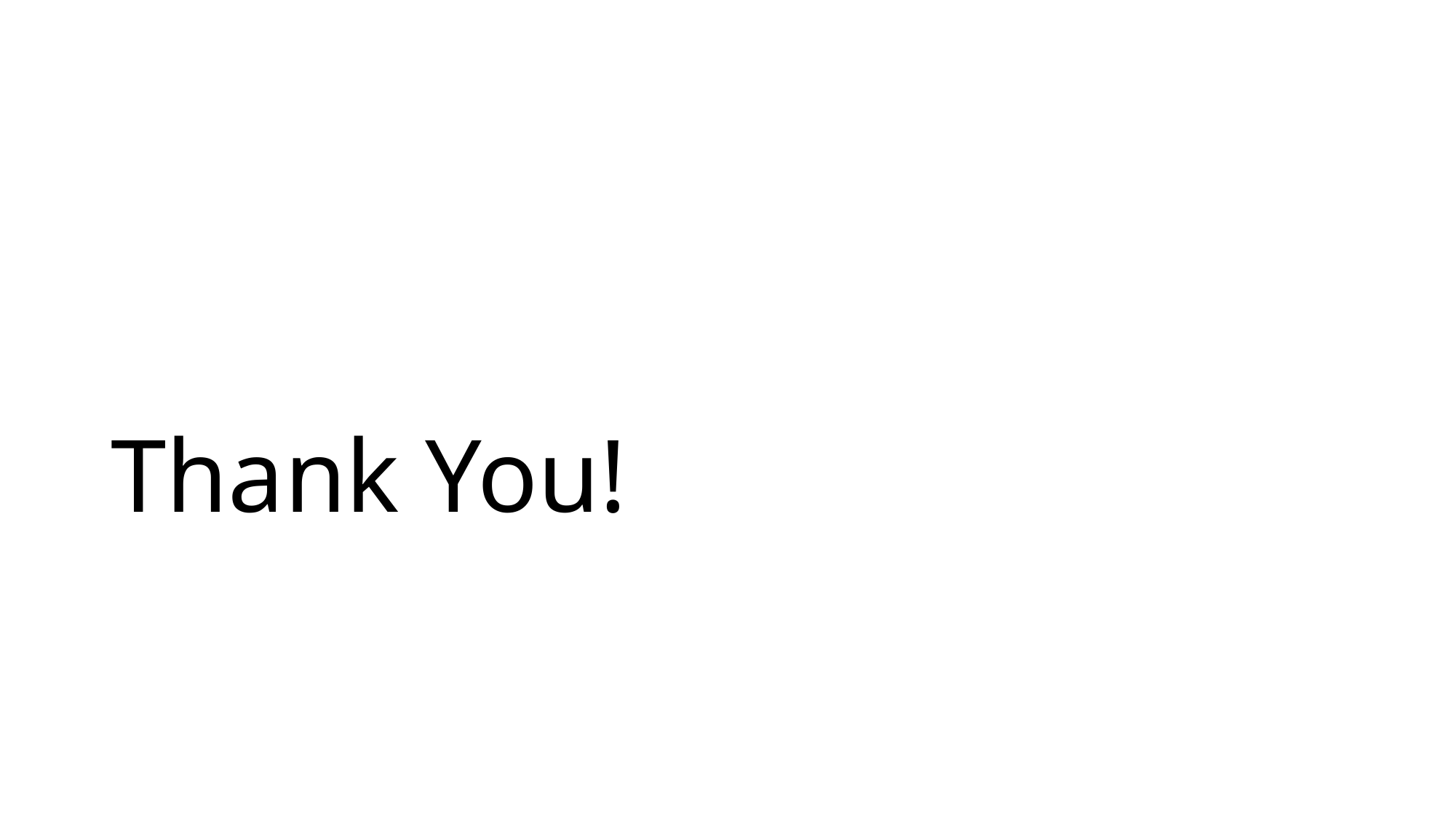

# Thank You!
