# Supplementary material for: What/Why/When/Where/How Framework and Faculty Development Workshop to Improve the Utility of Narrative Evaluations for Assessing Internal Medicine Residents
Source: MedEdPORTAL. 2024 Jul 30;20:11420. doi: 10.15766/mep_2374-8265.11420 (PMC11286767; doi:10.15766/mep_2374-8265.11420)
Supplement: Supplementary file 1 — Workshop Slides.pptxFramework.docxMock Learner Video 1.mp4Mock Learner Video 2.mp4Surveys.docxUtility Grading Rubric.docxFacilitator Guide.docx [file mep_2374-8265.11420-s001.zip › B. Framework.docx]

Appendix B: Narrative Comment Framework

This framework will be described in detail during the workshop. This figure can be used as a reference for your participants during the workshop and to use as a reference card when writing narrative evaluations in the future.
