# Supplementary material for: What/Why/When/Where/How Framework and Faculty Development Workshop to Improve the Utility of Narrative Evaluations for Assessing Internal Medicine Residents
Source: MedEdPORTAL. 2024 Jul 30;20:11420. doi: 10.15766/mep_2374-8265.11420 (PMC11286767; doi:10.15766/mep_2374-8265.11420)
Supplement: Supplementary file 1 — Workshop Slides.pptxFramework.docxMock Learner Video 1.mp4Mock Learner Video 2.mp4Surveys.docxUtility Grading Rubric.docxFacilitator Guide.docx [file mep_2374-8265.11420-s001.zip › _Educational Summary Report_11420.pdf]

MedEdPORTAL® The AAMC Journal of Teaching and Learning Resources

Original Publication Open Access

What/Why/When/Where/How Framework and Faculty Development Workshop to Improve the Utility of Narrative Evaluations for Assessing Internal Medicine Residents

Dheepa R. Sekar, MD*, Kristen Ann Ehrenberger, MD, PhD, Allie Dakroub, MD, MS, Scott Rothenberger, PhD, Thomas Grau, MD, Andrea E. Carter, MD, MS

*Corresponding author: dsekar2@emory.edu

Abstract

Introduction: Clinical competency committees (CCCs) rely on narrative evaluations to assess resident competency. Despite the emphasis on these evaluations, their utility is frequently hindered by lack of sufficient detail for use by CCCs. Prior resources have sought to improve specificity of comments and use of evaluations by residents but not their utility for CCCs in assessing trainee performance. **Methods:** We developed a 1-hour faculty development workshop focused on a newly devised framework for Department of Medicine faculty supervising internal medicine residents. The what/why/when/where/how framework highlighted key features of useful narrative evaluations: behaviors of strength and growth, contextualized observations, improvement over time, and actionable next steps. Workshop sessions were implemented at a large multisite internal medicine residency program. We assessed the workshop by measuring attendee confidence and skill in writing narrative evaluations useful for CCCs. Skill was assessed through a rubric adapted from literature on the utility of narrative evaluations. **Results:** Fifty-four participants started the presurvey, and 33 completed the workshop, for a response rate of 61%. Participant confidence improved pre-, post-, and 3 months postworkshop. Total utility scores improved in mock evaluations from 12.4 to 15.5 and in real evaluations from 13.7 to 15.0, but only some subcomponent scores improved, with fewer improving in the real evaluations. **Discussion:** A short workshop focusing on our framework improves confidence and utility of narrative evaluations of internal medicine residents for use by CCCs. Next steps should include developing more challenging components of narrative evaluations for continued improvement in trainee performance and faculty assessment.

Keywords
Clinical Competency Committee, Narrative Evaluations, Assessment, Competency-Based Medical Education (Competencies, Milestones, EPAs), Faculty Development, Internal Medicine

Educational Objectives

By the end of this workshop, participants will be able to:

1. Describe important features of a useful narrative evaluation.
2. Apply the what/why/when/where/how framework to guide observations of residents.
3. Examine sample narrative evaluations for features of a useful narrative evaluation.
4. Formulate a useful narrative evaluation by applying the what/why/when/where/how framework.

Citation:
Sekar DR, Ehrenberger KA, Dakroub A, Rothenberger S, Grau T, Carter AE. What/why/when/where/how framework and faculty development workshop to improve the utility of narrative evaluations for assessing internal medicine residents. *MedEdPORTAL*. 2024;20:11420. https://doi.org/10.15766/mep_2374-8265.11420

Introduction

Evaluation of trainees has undergone extensive revision by the Accreditation Council for Graduate Medical Education (ACGME) with the adoption of six core competencies in 2001 and developmentally based, specialty-specific educational milestones in 2013.[1,2] With these changes came the requirement that each training program establish a clinical competency committee (CCC), whose charge is to ensure trainees achieve competence for independent practice by the time of graduation as well as demonstrating ongoing development over the course of training.[1] Most CCCs rely on multiple mediums to inform assessment of residents along this developmental trajectory, but the most valued source is evaluation comments from end-of rotation

Copyright © 2024 Sekar et al. This is an open-access publication distributed under the terms of the Creative Commons Attribution license.

evaluations forms completed by faculty who directly supervise residents in the clinical setting.[3,4]

End-of-rotation evaluation forms vary in ability to capture resident performance. While attempts have been made to optimize the scales for measuring resident performance, they do not always reflect the nuances of resident performance, making narrative comments essential.[3,5-8] In fact, comments alone can accurately rank order the performance of learners.[9] Narrative comments are crucial for CCCs to provide trainees with steps for improvement, so it is important to improve the quality and utility of written comments for use by CCCs.[5-8,10]

The literature describes high-quality written evaluations as being specific, with contextualized examples.[6,10] However, faculty face high cognitive load in converting observations to written comments as they try to recall examples or consider which might be useful in reflecting resident competency.[6,9,11] Several studies have attempted to improve the quality of written evaluations. These studies have looked at improvement in the context of resident reception of feedback, specificity of comments provided, and overall quality of narrative comments.[7,12-17] To our knowledge, no previous resources have been created with the goal of improving the utility of written comments for use by CCCs.

We implemented and evaluated a faculty development workshop aimed at improving the utility of narrative comments for use by the CCC at our internal medicine residency program.

Methods

Setting and Participants

We implemented and evaluated a 1-hour workshop for faculty with the goal of improving the utility of narrative comments for use by CCCs. We delivered the workshop at standing faculty development sessions in four divisions within the Department of Medicine at the University of Pittsburgh Medical Center in May-August 2021. All faculty who regularly supervised internal medicine residents were invited via email to attend. Participation in the workshop was entirely voluntary. We created the workshop to be delivered both in person and virtually. A 1-hour interactive adaptable workshop model was chosen for feasibility in reaching a large faculty body located at multiple sites in multiple divisions of varying clinical structures.

Workshop

The workshop was an interactive didactic (Appendix A) that focused on the use of a what/why/when/where/how framework (Appendix B) that not only consolidated the core features that CCC members used in evaluating residents but also decreased cognitive load of observing faculty.

Workshop content focused on (1) the value of narrative comments, (2) actionable feedback as described in Kim Scott's book *Radical Candor,*[18] and (3) key features of useful narrative comments using a what/why/when/where/how framework. The workshop demonstrated the framework through step-by-step analysis of examples of useful and not useful narrative comments. The framework was informed by a local formal qualitative needs assessment of our CCC, which highlighted the usefulness of comments on behavioral content, performance relative to peers, and next steps for improvement in assessing residents. These concepts were also highlighted in the literature that we incorporated in developing the what/why/when/where/how framework. The "What behaviors did you observe?" and "Why are these behaviors reflective of their performance?" components emphasized the importance of including specific behavioral examples and reflecting on why these behaviors were salient representations of performance.[19] The "When in training?" and "Where were the behaviors observed?" components emphasized the contexts of the observation, allowing observing faculty to provide comparisons to a mental scale.[20] The "How can the trainee improve?" component emphasized the importance of providing next steps for improvement in guiding the resident for continued improvement.[20]

Participants were asked to apply the framework to mock learner videos. We created two 5-minute mock learner videos (Appendices C and D) that captured typical skills of senior internal medicine residents during an inpatient general medicine service. Participants wrote mock evaluations (MEs) immediately prior to the workshop, which demonstrated priming, and immediately after the workshop using our framework, which demonstrated deliberate practice of the new skill.

Outcomes Measured

We evaluated effects of the workshop on (1) participants' confidence in writing narrative comments, (2) utility of participants' narrative comments on mock resident cases, and (3) utility of participants' narrative comments on real end-of-rotation resident evaluations in MedHub.

Confidence survey: We assessed confidence through electronic Qualtrics XM surveys (Appendix E) prior to, after, and 3 months after the workshop. Survey question responses were based on a 5-point Likert scale (1 = *not at all confident*, 5 = *very confident*). Each item was developed and iteratively refined with input from CCC members and observing faculty.

Copyright © 2024 Sekar et al. This is an open-access publication distributed under the terms of the Creative Commons Attribution license.

Utility of comments on mock resident cases: We assessed skill development by evaluating utility of comments on mock resident cases. Participants submitted a narrative ME of the learner case prior to and after the workshop. We deidentified and randomized the pre- and postworkshop MEs. Two medicine-pediatric CCC members graded each ME for its utility in providing useful information for the CCC using the utility grading rubric (Appendix F). We chose CCC members outside of the internal medicine residency program to allow for generalizability of the content, and we specifically chose medicine-pediatric CCC members for similarity of resident milestones of this discipline to internal medicine. We used the average of the two graders' item scores and total scores in the final analysis.

Utility of comments on real resident evaluations: We assessed practice change by evaluating the utility of narrative comments written by participants on end-of-rotation MedHub real evaluation (RE) forms routinely collected by the residency program pre- and postworkshop. MedHub evaluations with no written comments were excluded. We performed deidentification, randomization, and scoring as described above for MEs. Participants were not informed of the time frame in which postworkshop narrative evaluations would be assessed in an attempt to decrease the likelihood of a Hawthorne effect.

Methods of Analysis

Development of utility grading rubric: To assess utility of narrative evaluations for use by the CCC, we created a grading rubric (Appendix F) modeled after a previously published grading rubric for assessing the overall quality of narrative evaluations.[21] Our grading rubric included an overall score as well as components of the framework: behaviors of strength, behaviors of growth, context, improvement over time, and actionable next steps. The items and scale of the adapted graded rubric were discussed and iteratively optimized with a focus group of four members of the internal medicine CCC, resulting in six items rated on a 5-point Likert scale. Then, two medicine-pediatric residency CCC members scored six randomly selected sample narrative evaluations and discussed for consensus. This discussion resulted in the final grading rubric with the same six items but now rated on a 4-point Likert scale (1 = *not useful,* 4 = *extremely useful*), for a total score of 24. We assessed the rubric for interrater reliability using interclass correlation coefficients based on all analyzed MEs.

*Statistical analysis:*We assessed changes in confidence pre-, post-, and 3 months postworkshop for each confidence item using a mixed-effects linear regression model fit with time as the categorical predictor variable. Bonferroni adjustments were employed when analyzing pairwise differences between time points. The evaluation utility overall score and subdomains on both the MEs and REs were also analyzed using mixed-effects linear regression models. We chose this modeling approach to account for repeated measures per participant and make maximum use of all available data. We used Stata (StataCorp) for statistical analysis and Excel for data management, deidentification, and randomization where needed, and a significance level of 5% was assumed.

Facilitator Guide

We include here a guide for facilitation of the workshop (Appendix G).

This project was designated exempt by the University of Pittsburgh Institutional Review Board.

Results

Participants

Fifty-four participants started the presurvey, and 33 completed the workshop, for a response rate of 61%. The participants represented varying time on faculty, experience on the CCC, and several subspecialities (Table 1).

Utility Grading Rubric

The interclass correlation for interrater reliability for the total score on the utility grading rubric (Appendix F) was .83.

Confidence Survey

The 33 faculty who participated in the workshop and completed both pre- and postsurveys were included in the confidence analysis. All confidence items significantly improved pre- to postworkshop and sustained at 3 months: overall confidence (2.7 to 3.6 to 3.5), confidence in writing narrative evaluations

Table 1. Characteristics of Participants (*N* = 33)

Characteristic	No.	%
Prior training	5	15
Years on faculty		
0-4	14	42
5-10	4	12
>10	6	18
Clinical competency committee experience	9	27
Subspecialty		
General internal medicine	13	39
Hospitalist	7	21
Infectious diseases	6	18
Palliative care	2	6
Cardiology	1	3
Pediatric critical care medicine	1	3
Geriatrics	1	3

Copyright © 2024 Sekar et al. This is an open-access publication distributed under the terms of the Creative Commons Attribution license.

useful for the resident (2.7 to 3.6 to 3.5) and program leadership (2.8 to 3.8 to 3.6), and confidence in providing specific features: specific behaviors (2.8 to 3.6 to 3.7), context (2.8 to 3.8 to 3.6), and improvement over time (2.8 to 3.6 to 3.7), with $p < .05$ for all comparisons between pre- and postworkshop and pre- and 3 months postworkshop (Table 2). There was no significant difference between immediate postworkshop and 3 months postworkshop for all comparisons.

Utility of comments on mock resident cases: Thirty-seven MEs were submitted preworkshop, and 33 MEs were submitted postworkshop. The total utility score of learner case narrative evaluations significantly improved postworkshop from 12.4 to 15.5, with $p < .001$. Scores for specific behaviors of strength, specific behaviors of growth, context, and overall scores significantly improved. Scores for improvement over time and actionable next steps did not significantly improve (Table 3).

Utility of comments on real resident evaluations: Of the 33 participants who completed the workshop, 17 participants had REs both pre- and postworkshop that met inclusion criteria. From these 17 participants, we collected 128 REs preworkshop and 95 REs postworkshop. We randomly selected up to five evaluations for each participant for each pre- and postworkshop for the final analysis. Three postworkshop REs did not include a narrative comment and were excluded. The final analysis included a total of 73 preworkshop and 58 postworkshop REs.

The mean total utility score was 13.7 preworkshop and 15.0 postworkshop, with $p < .05$. Context significantly improved from 2.0 preworkshop to 2.6 postworkshop, with $p < .001$, but there was no significant improvement in the other domains. The overall utility score was 2.6 preworkshop and 2.8 postworkshop, with $p = .07$ (Table 4).

Discussion

This 1-hour faculty development workshop to improve the utility of narrative evaluations in assessing internal medicine residents was designed for feasibility in impactfully reaching a broad audience through a short session focused on decreasing cognitive load. This framework and deliberate practice led to improved confidence, skill, and behavioral practice of faculty in writing narrative comments on internal medicine residents that would be useful for CCCs.

The workshop's design focused on capturing behavioral details of resident performance in narrative comments through a framework. The framework built upon prior resources that also focused on behavioral examples. Prior resources used a process flow or a reference card for competencies but not specific descriptors of behaviors useful for CCCs.[7,14] We highlighted the key features of behaviors CCCs use in assessing residents that had not been captured in previous resources, namely, the components of when, where, and how/next steps.[7,13,14] Additionally, the framework addressed concerns of faculty time and cognitive load by providing an anchor for these details and a reference tool to use in practice. Another study provided direct in-person feedback to faculty on their narrative comments, which can be time consuming to implement.[12] This workshop relied on an easily distributable framework to guide narrative writing. Furthermore, the workshop's evaluation addressed an important question about the utility of narrative comments for the purpose of resident evaluation. Prior studies evaluated syntax for specificity or resident reception, but it was unclear if CCCs found them more useful.[13,14] In the assessment of this workshop, we used a scale of utility and demonstrated local content, face validity, and interrater reliability. This scale can be used by other programs

Table 2. Confidence in Writing Narrative Evaluations

Confidence Survey Item[a]	*M* (95% CI)			*p*		
	Preworkshop	Postworkshop	3 Months Postworkshop	Pre- vs. Postworkshop	Pre- vs. 3 Months Postworkshop	Post- vs. 3 Months Postworkshop
Overall confidence in writing narrative evaluations	2.7 (2.5 to 2.9)	3.6 (3.4 to 3.9)	3.5 (3.1 to 3.8)	<.001	<.001	.98
Confidence in writing narrative evaluations for resident	2.7 (2.5 to 2.9)	3.6 (3.3 to 3.8)	3.5 (3.1 to 3.9)	<.001	<.001	>.99
Confidence in writing narrative evaluations for leadership	2.8 (2.6 to 3.0)	3.8 (3.5 to 4.0)	3.6 (3.2 to 4.0)	<.001	.001	>.99
Confidence in providing specific behaviors	2.8 (2.6 to 3.0)	3.6 (3.4 to 3.9)	3.7 (3.4 to 4.1)	<.001	<.001	>.99
Confidence in providing appropriate context	2.8 (2.6 to 3.0)	3.8 (3.5 to 4.1)	3.6 (4.2 to 4.0)	<.001	.001	.90
Confidence in providing change in resident performance over time	2.8 (2.5 to 3.0)	3.6 (3.3 to 3.8)	3.7 (2.2 to 4.1)	<.001	<.001	>.99

[a]Rated on a 5-point Likert scale (1 = *not at all confident*, 5 = *very confident*).

Copyright © 2024 Sekar et al. This is an open-access publication distributed under the terms of the Creative Commons Attribution license.

Table 3. Utility of Mock Evaluations

Rubric Item[a]	*M* (95% CI) Preworkshop	Postworkshop	Difference (95% CI)	*p*
Behaviors of strength	2.5 (2.2 to 2.8)	3.1 (2.8 to 3.4)	0.60 (0.20 to 1.00)	.003
Behaviors of growth	2.8 (2.6 to 3.1)	3.6 (3.4 to 3.8)	0.80 (0.50 to 1.00)	<.001
Context	1.6 (1.4 to 1.7)	2.2 (2.0 to 2.4)	0.60 (0.40 to 0.90)	<.001
Improvement over time	1.1 (1.0 to 1.2)	1.1 (1.0 to 1.2)	0.03 (−0.10 to 0.20)	.73
Actionable next steps	2.0 (1.7 to 2.4)	2.4 (2.1 to 2.8)	0.40 (−0.10 to 0.90)	.10
Overall	2.4 (2.2 to 2.6)	3.1 (2.8 to 3.3)	0.60 (0.40 to 0.90)	<.001
Total score	12.4 (11.5 to 13.3)	15.5 (14.5 to 16.4)	3.10 (2.00 to 4.20)	<.001

[a]Rated on a 4-point Likert scale (1 = *not useful*, 4 = *extremely useful*).

attempting to assess local narrative comments for utility for their CCCs.

Not all subcomponents of the narrative comments improved in the MEs and REs. In the MEs, improvement over time and next steps did not improve. These components require more depth of reflection on resident performance and may also require additional faculty training. Additionally, the level of detail in the mock videos may not have been sufficient to accurately capture this skill. In the REs, only the context subcomponent improved significantly. Context of time in training and patient acuity may be simpler concepts to report. The assessment of REs shows application in both a different and a delayed setting, which may have led to loss in some detail of the skill.

An important limitation of this project is that we did not specifically assess the role of bias in narrative evaluations. Several studies have shown that the use of language changes based on reported gender and race,[22,23] and this would be an interesting area for future study. Other limitations include delivery and assessment at a single large academic institution in a specific residency specialty, so results may not be generalizable across other institutions or specialties. Participation was voluntary across many divisions, but participation outside of general internal medicine and hospital medicine was limited. Additionally, voluntary participation may have introduced self-selection bias as participants could have been more likely to use workshop concepts in future narrative evaluations than others would. Confidence surveys were based on Likert scales, which might have introduced response bias. We are unable to comment on the effect of this workshop compared to a broader baseline including faculty who did not participate. In addition, writing narrative evaluations using the what/why/when/where/how framework may take more time and effort than traditional narrative evaluations. Finally, while the scoring tool was adapted from a previously published rubric, was reviewed and revised by local content experts, and was assessed for interrater reliability, its use may require further reliability and validity testing before application in other settings.

Next steps for continued improvement through use of the workshop's content and framework may require repetition of practice. Addressing this gap could be as simple as providing the framework as a reference at the beginning of each rotation, as a reference in the evaluation form itself, or both. Another important next step is to assess whether this framework's focus on objective comments helps decrease gender or racial bias in evaluations. If programs are able to incorporate the framework into learning management software, building in components that flag biased language may mitigate overall bias in narrative evaluations. Such an implementation would require careful assessment and validation. Additionally, this

Table 4. Utility of Real Evaluations

Rubric Item[a]	*M* (95% CI) Preworkshop	Postworkshop	Difference (95% CI)	*p*
Behaviors of strength	3.1 (3.0 to 3.2)	3.2 (3.1 to 3.3)	0.10 (−0.02 to 0.20)	.12
Behaviors of growth	2.2 (1.9 to 2.4)	2.2 (1.9 to 2.5)	0.03 (−0.30 to 0.40)	.88
Context	2.0 (1.7 to 2.3)	2.6 (2.3 to 2.9)	0.60 (0.30 to 0.90)	<.001
Improvement over time	1.5 (1.3 to 1.7)	1.6 (1.4 to 1.8)	0.20 (−0.20 to 0.40)	.33
Actionable next steps	2.4 (2.0 to 2.7)	2.5 (2.1 to 2.9)	0.10 (−0.20 to 0.50)	.42
Overall	2.6 (2.4 to 2.8)	2.8 (2.5 to 3.0)	0.20 (−0.01 to 0.40)	.07
Total score	13.7 (12.6 to 14.8)	15.0 (13.8 to 16.1)	1.20 (0.20 to 2.20)	.02

[a]Rated on a 4-point Likert scale (1 = *not useful*, 4 = *extremely useful*).

Copyright © 2024 Sekar et al. This is an open-access publication distributed under the terms of the Creative Commons Attribution license.

workshop relies on individual reflection on content and skills. The focus on individual reflection can be improved by allowing participants to apply the utility rubric to their evaluations as a form of ongoing formative self-feedback. Future iterations could be further improved by including small-group discussions of mock learner videos, allowing participants to learn from each other's observations and reflections, or employing a mock CCC approach. Future implementations could also provide regular application of the utility rubric to REs for ongoing formative feedback to faculty as well as assessment of the workshop's long-term impact. Finally, future implementations should assess whether subsequent interactive sessions are needed or if a reference to the framework will provide a similar effect but allow for maintained feasibility for busy academic faculty.

Overall, this short workshop provides a high-yield framework resulting in improved overall utility of real narrative comments for use by CCCs. The workshop fulfills the needs of two important stakeholders in narrative comments—CCCs and observing faculty—by addressing inconsistent views and providing a simple high-yield framework. While the workshop was implemented at a single institution, its materials are translatable to training programs with CCCs that use a developmental approach.

Appendices

A. Workshop Slides.pptx
B. Framework.docx
C. Mock Learner Video 1.mp4
D. Mock Learner Video 2.mp4
E. Surveys.docx
F. Utility Grading Rubric.docx
G. Facilitator Guide.docx

All appendices are peer reviewed as integral parts of the Original Publication.

Dheepa R. Sekar, MD: Assistant Professor, Division of General Internal Medicine and Geriatrics, Department of Medicine, Emory University School of Medicine; ORCID: https://orcid.org/0000-0001-8871-0100

Kristen Ann Ehrenberger, MD, PhD: Assistant Professor, Division of General Internal Medicine, Department of Medicine and Department of Pediatrics, University of Pittsburgh School of Medicine

Allie Dakroub, MD, MS: Assistant Professor, Division of General Internal Medicine, Department of Medicine and Department of Pediatrics, University of Pittsburgh School of Medicine

Scott Rothenberger, PhD: Assistant Professor, Division of General Internal Medicine, Department of Medicine, University of Pittsburgh School of Medicine

Thomas Grau, MD: Associate Professor, Division of General Internal Medicine, Department of Medicine, University of Pittsburgh School of Medicine; Associate Chief of Staff of Education, VA Pittsburgh Healthcare System

Andrea E. Carter, MD, MS: Assistant Professor, Division of General Internal Medicine, Department of Medicine, University of Pittsburgh School of Medicine

Disclosures
None to report.

Funding/Support
None to report.

Prior Presentations
Sekar D, Ganti A, Memari M, Carter A. From vague comments to actionable feedback: train your faculty to write high-quality end-of-rotation written evaluations of trainees. Workshop presented virtually at: Society for General Internal Medicine Mid-Atlantic Regional Meeting; October 28-29, 2021.

Sekar D, Ganti A, Memari M, Carter A. From vague comments to actionable feedback: train your faculty to write high-quality end-of-rotation written evaluations of trainees. Workshop presented at: Alliance for Academic Internal Medicine National Meeting; April 10, 2022; Charlotte, NC.

Ethical Approval
The University of Pittsburgh Institutional Review Board deemed further review of this project not necessary.

References

1. Andolsek K, Padmore J, Hauer KE, Ekpenyong A, Edgar L, Holmboe E. *Clinical Competency Committees: A Guidebook for Programs*. 3rd ed. Accreditation Council for Graduate Medical Education; 2020. Accessed June 17, 2024. https://www.acgme.org/globalassets/acgmeclinicalcompetencycommitteeguidebook.pdf
2. Nasca TJ, Philibert I, Brigham T, Flynn TC. The Next GME Accreditation System—rationale and benefits. *N Engl J Med.* 2012;366(11):1051-1056. https://doi.org/10.1056/NEJMsr1200117
3. Hemmer PA, Hawkins R, Jackson JL, Pangaro LN. Assessing how well three evaluation methods detect deficiencies in medical students' professionalism in two settings of an internal medicine clerkship. *Acad Med.* 2000;75(2):167-173. https://doi.org/10.1097/00001888-200002000-00016
4. Hauer KE, Chesluk B, Iobst W, et al. Reviewing residents' competence: a qualitative study of the role of clinical competency

Copyright © 2024 Sekar et al. This is an open-access publication distributed under the terms of the Creative Commons Attribution license.

committees in performance assessment. *Acad Med.* 2015;90(8):1084-1092. https://doi.org/10.1097/ACM.0000000000000736

5. Lurie SJ, Mooney CJ, Lyness JM. Measurement of the general competencies of the Accreditation Council for Graduate Medical Education: a systematic review. *Acad Med.* 2009;84(3):301-309. https://doi.org/10.1097/ACM.0b013e3181971f08
6. Ginsburg S, McIlroy J, Oulanova O, Eva K, Regehr G. Toward authentic clinical evaluation: pitfalls in the pursuit of competency. *Acad Med.* 2010;85(5):780-786. https://doi.org/10.1097/ACM.0b013e3181d73fb6
7. Holmboe ES, Fiebach NH, Galaty LA, Huot S. Effectiveness of a focused educational intervention on resident evaluations from faculty: a randomized controlled trial. *J Gen Intern Med.* 2001;16(7):427-434. https://doi.org/10.1046/j.1525-1497.2001.016007427.x
8. Crossley J, Jolly B. Making sense of work-based assessment: ask the right questions, in the right way, about the right things, of the right people. *Med Educ.* 2012;46(1):28-37. https://doi.org/10.1111/j.1365-2923.2011.04166.x
9. Ginsburg S, van der Vleuten CPM, Eva KW. The hidden value of narrative comments for assessment: a quantitative reliability analysis of qualitative data. *Acad Med.* 2017;92(11):1617-1621. https://doi.org/10.1097/ACM.0000000000001669
10. Green ML, Holmboe E. Perspective: the ACGME toolbox: half empty or half full? *Acad Med.* 2010;85(5):787-790. https://doi.org/10.1097/ACM.0b013e3181d737a6
11. Ginsburg S, van der Vleuten C, Eva KW, Lingard L. Hedging to save face: a linguistic analysis of written comments on in-training evaluation reports. *Adv Health Sci Educ Theory Pract.* 2016;21(1):175-188. https://doi.org/10.1007/s10459-015-9622-0
12. Dudek NL, Marks MB, Wood TJ, et al. Quality evaluation reports: can a faculty development program make a difference? *Med Teach.* 2012;34(11):e725-e731. https://doi.org/10.3109/0142159X.2012.689444
13. Hatala R, Sawatsky AP, Dudek N, Ginsburg S, Cook DA. Using in-training evaluation report (ITER) qualitative comments to assess medical students and residents: a systematic review. *Acad Med.* 2017;92(6):868-879. https://doi.org/10.1097/ACM.0000000000001506
14. Zelenski AB, Tischendorf JS, Kessler M, et al. Beyond "read more": an intervention to improve faculty written feedback to learners. *J Grad Med Educ.* 2019;11(4):468-471. https://doi.org/10.4300/JGME-D-19-00058.1
15. Sargeant J, Armson H, Driessen E, et al. Evidence-informed facilitated feedback: the R2C2 feedback model. *MedEdPORTAL.* 2016;12:10387. https://doi.org/10.15766/mep_2374-8265.10387
16. Johnson NR, Pelletier A, Royce C, et al. Feedback Focused: a learner- and teacher-centered curriculum to improve the feedback exchange in the obstetrics and gynecology clerkship. *MedEdPORTAL.* 2021;17:11127. https://doi.org/10.15766/mep_2374-8265.11127
17. Bhavaraju V, Bartlett K, Schumacher D, Guillot A. Faculty development series on assessment in graduate medical education: the Milestone Project. *MedEdPORTAL.* 2014;10:9898. https://doi.org/10.15766/mep_2374-8265.9898
18. Scott K. *Radical Candor: Be a Kick-Ass Boss Without Losing Your Humanity*. St. Martin's Press; 2017.
19. Dudek N, Dojeiji S. Twelve tips for completing quality in-training evaluation reports. *Med Teach.* 2014;36(12):1038-1042. https://doi.org/10.3109/0142159X.2014.932897
20. Holmboe ES, Yamazaki K, Hamstra SJ. The evolution of assessment: thinking longitudinally and developmentally. *Acad Med.* 2020;95(11)(suppl):S7-S9. https://doi.org/10.1097/ACM.0000000000003649
21. Dudek NL, Marks MB, Wood TJ, Lee AC. Assessing the quality of supervisors' completed clinical evaluation reports. *Med Educ.* 2008;42(8):816-822. https://doi.org/10.1111/j.1365-2923.2008.03105.x
22. Klein R, Snyder ED, Koch J, et al. Analysis of narrative assessments of internal medicine resident performance: are there differences associated with gender or race and ethnicity? *BMC Med Educ.* 2024;24:72. https://doi.org/10.1186/s12909-023-04970-2
23. Rojek AE, Khanna R, Yim JWL, et al. Differences in narrative language in evaluations of medical students by gender and under-represented minority status. *J Gen Intern Med.* 2019;34(5):684-691. https://doi.org/10.1007/s11606-019-04889-9

Received: November 9, 2023
Accepted: March 14, 2024
Published: July 30, 2024

Copyright © 2024 Sekar et al. This is an open-access publication distributed under the terms of the Creative Commons Attribution license.
